# Unsupervised physiological noise correction of functional magnetic resonance imaging data using phase and magnitude information (PREPAIR)

**DOI:** 10.1002/hbm.26152

**Published:** 2022-11-19

**Authors:** David Bancelin, Beata Bachrata, Saskia Bollmann, Pedro de Lima Cardoso, Pavol Szomolanyi, Siegfried Trattnig, Simon Daniel Robinson

**Affiliations:** ^1^ High Field MR Centre, Department of Biomedical Imaging and Image‐guided Therapy Medical University of Vienna Vienna Austria; ^2^ Karl Landsteiner Institute for Clinical Molecular MR in Musculoskeletal Imaging Vienna Austria; ^3^ Centre for Advanced Imaging The University of Queensland Brisbane Australia; ^4^ Department of Neurology Medical University of Graz Graz Austria

**Keywords:** PREPAIR, fMRI, phase data, physiological noise, noise correction, unsupervised

## Abstract

Of the sources of noise affecting blood oxygen level‐dependent functional magnetic resonance imaging (fMRI), respiration and cardiac fluctuations are responsible for the largest part of the variance, particularly at high and ultrahigh field. Existing approaches to removing physiological noise either use external recordings, which can be unwieldy and unreliable, or attempt to identify physiological noise from the magnitude fMRI data. Data‐driven approaches are limited by sensitivity, temporal aliasing, and the need for user interaction. In the light of the sensitivity of the phase of the MR signal to local changes in the field stemming from physiological processes, we have developed an unsupervised physiological noise correction method using the information carried in the phase and the magnitude of echo‐planar imaging data. Our technique, Physiological Regressor Estimation from Phase and mAgnItude, sub‐tR (PREPAIR) derives time series signals sampled at the slice TR from both phase and magnitude images. It allows physiological noise to be captured without aliasing, and efficiently removes other sources of signal fluctuations not related to physiology, prior to regressor estimation. We demonstrate that the physiological signal time courses identified with PREPAIR agree well with those from external devices and retrieve challenging cardiac dynamics. The removal of physiological noise was as effective as that achieved with the most used approach based on external recordings, RETROICOR. In comparison with widely used recording‐free physiological noise correction tools—PESTICA and FIX, both performed in unsupervised mode—PREPAIR removed significantly more respiratory and cardiac noise than PESTICA, and achieved a larger increase in temporal signal‐to‐noise‐ratio at both 3 and 7 T.

## Graphical abstract

We have developed an unsupervised physiological noise correction method using the information carried in the phase and the magnitude of echo‐planar imaging data. We demonstrate that the physiological signal time courses identified with Physiological Regressor Estimation from Phase and mAgnItude, sub‐tR (PREPAIR) not only agree well with those from external devices, but also retrieve challenging cardiac dynamics.

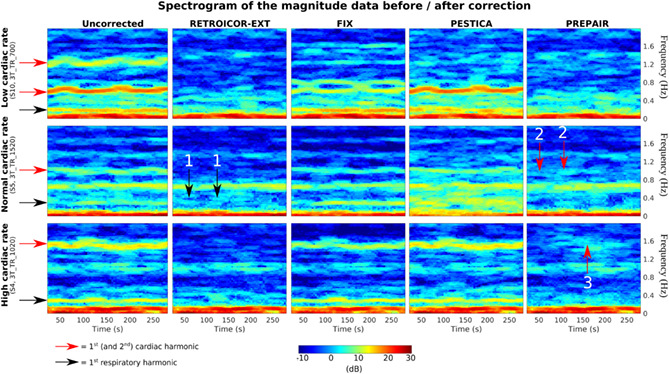

## 1 INTRODUCTION

Functional magnetic resonance imaging (fMRI) using the blood oxygen level‐dependent (BOLD) (Ogawa et al., 1990) contrast has become the most powerful and ubiquitous method to investigate brain function during task processing and at rest. BOLD signals are corrupted, however, by non‐white noise arising from scanner instability, head motion and physiological fluctuations related to the cardiac and respiratory cycles (Caballero‐Gaudes & Reynolds, 2017; Liu, 2016; Lund et al., 2006; Reynaud et al., 2017). These decrease the temporal signal‐to‐noise‐ratio (tSNR), limit the tSNR gain associated with increasing static magnetic field (Triantafyllou et al., 2005) and reduce sensitivity to BOLD activation (Biswal et al., 1996; Lund et al., 2006).

Respiration and cardiac‐related fluctuations in echo‐planar imaging (EPI)‐based BOLD fMRI are responsible for most of the variance in gray matter and cerebrospinal fluid (CSF) (Bianciardi et al., 2009; Jezzard et al., 1993; Weisskoff et al., 1993; Windischberger et al., 2002). Susceptibility variations during breathing engender small changes in the magnetic field which lead to artifacts in the BOLD signal (Raj et al., 2001), as do signals related to the chest moving at the respiration frequency, which also translates into head motion (Birn et al., 2008; Krüger & Glover, 2001). Moreover, the pulsatile nature of the cardiac cycle causes variations in the blood flow as well as tissue movements which can contaminate the measured MR signal in voxels containing major blood vessels (Dagli et al., 1999), while the brainstem is particularly affected by physiological noise due to its proximity to the fourth ventricle and arteries (Beissner et al., 2011; Harvey et al., 2008; Matt et al., 2019).

Several methods have been developed to remove physiological perturbations and increase statistical power and statistical validity. Filtering approaches (Biswal et al., 1996; Chuang & Chen, 2001) and statistical models (Agrawal et al., 2020) perform well with fast fMRI but can fail when the frequency of the physiological noise exceeds the Nyquist rate, which is generally $2/{\mathrm{TR}}_{\mathrm{vol}}$, where ${\mathrm{TR}}_{\mathrm{vol}}$ is the volume repetition time. Despite the more rapid acquisition allowed by simultaneous multi‐slice (SMS) EPI (Barth et al., 2016; Feinberg et al., 2010; Feinberg & Setsompop, 2013; Setsompop et al., 2012), ${\mathrm{TR}}_{\mathrm{vol}}$ is still generally in excess of what would be needed to critically sample cardiac fluctuations, which is, for example, 400 ms for a heart rate of 75 bpm. However, slice time series can be reordered into temporal rather than spatial order to critically sample physiological noise (Frank et al., 2001). Alternative techniques involve the simultaneous acquisition of physiological signals during the fMRI experiment. RETROICOR (Glover et al., 2000; Harvey et al., 2008) models physiological noise using Fourier expansions of the physiological recordings while other approaches model the respiration and cardiac response functions of lower frequency fluctuations (Birn et al., 2006; Birn et al., 2008; Chang et al., 2009). Yet, many MR sequences do not allow easy integration of physiological measurements. For instance, this is currently impossible with Siemens multiband EPI sequences. An alternative solution for recording traces on the scanner exist (e.g., for Siemens' scanners, based on commands issued to the Physiological Monitoring Unit [PMU] via telnet) but this also requires coordination of the signal trace with the dicom images and trigger events from the scanner, which can be problematic. Besides, external recordings increase the time overhead in subject handling and can be unreliable if the breathing belt becomes loose or slips (leading to signal loss) or is too tight (leading to a saturation in values) (Kasper et al., 2017), or, in the case of photoplethysmography, if noise (e.g., high‐frequency, power‐line interference, baseline drift, motion artifact) corrupt the signal (Elgendi, 2012). Databases like the Human Connectome Project (HCP) (Glasser et al., 2013) also contain corrupted physiological recordings which can require manual screening of the physiological noise signals (Aslan et al., 2019).

As physiological fluctuations are clearly identified in specific anatomical regions of the brain, many authors have explored the possibility of isolating cardiac and respiration signals directly from the fMRI data. Most common unsupervised data‐driven methods generate physiological regressors using component analysis techniques (Behzadi et al., 2007; Beissner et al., 2014; Churchill & Strother, 2013; Perlbarg et al., 2007) of noise regions of interests (ventricles, brainstem, and major blood vessels or in the CSF and white matter) or the whole brain (Beall, 2010; Beall & Lowe, 2007; Salimi‐Khorshidi et al., 2014; Thomas et al., 2002) to identify voxels dominated by physiological noise. For example, PESTICA (Beall, 2010; Beall & Lowe, 2007; Shin et al., 2016) correlates temporal‐ICA components with a prior spatial physiological noise distribution to extract the component with highest correlation in each slice. By reordering the selected components into a single time series (Frank et al., 2001), PESTICA increases the sampling rate, enabling the recovery of aliased signals. Other methods like FIX (Salimi‐Khorshidi et al., 2014) use prior information in existing trained datasets to automatically classify spatial‐ICA components as noise, or can be trained through by‐hand classification to improve performance. This process, and the need to check that the components identified by FIX as noise, makes the process onerous in studies with large numbers of subjects.

Most widely used physiological noise removal techniques only use magnitude fMRI data. However, data‐driven physiological correction stands to benefit from the use of phase information, as the phase of the MR signal is highly sensitive to the changes in *B*_0_ which accompany respiration (Hagberg et al., 2008; Hagberg et al., 2012; Petridou et al., 2009). Phase images are not generally saved in fMRI experiments, but this information is available as the inherent counterpart of the magnitude in the complex MRI data and can be reconstructed and used for physiological noise correction. Indeed, there is a growing interest in the information carried by the phase in fMRI to increase the statistical power in fMRI analysis (Rowe, 2005), for dynamic distortion correction (Dymerska et al., 2018; Robinson et al., 2021), quantitative susceptibility mapping (QSM) (Sun & Wilman, 2015), and functional QSM (Balla et al., 2014).

Using EPI phase poses some challenges, however: the need to find a solution to the phase‐sensitive combination of RF coil signals, particularly at ultrahigh field (Robinson et al., 2017), to unwrap phase images quickly and effectively and to remove unwanted signals from sources such as the cold‐head helium pump which affect the phase signal to a larger extent than the magnitude (Hagberg et al., 2012). While a small number of methods have made limited use of phase—to identify Gaussian‐distributed independent and identically distributed noise (Vizioli et al., 2021), for respiration (Cheng & Li, 2010; Le & Hu, 1996; Zahneisen et al., 2014), combined with ICA (Curtis & Menon, 2014)—none has taken full advantage of the sensitivity of phase to physiological fluctuations in a correction method for both respiratory and cardiac noise.

We propose a method which leverages phase and magnitude information, and obviates the need for external physiological signals and user interaction. This unsupervised, physiological data‐free and automatic Physiological Regressor Estimation from Phase and mAgnItude sub‐tR (PREPAIR) approach reveals both respiration and cardiac signals present in the fMRI time series for a wide range of TRs despite cardiac fluctuations being—considering the volume TR—critically sampled or undersampled. We adopt recently developed approaches to coil combination (Eckstein et al., 2018) and unwrapping (Dymerska et al., 2021) combined with temporal reordering and slice filtering to derive physiological signals sampled at the slice repetition interval rather than the volume repetition interval. High‐frequency fluctuations due to breathing motion and cardiac pulsatility are identified via nuisance signal variation. PREPAIR is compared with three commonly used physiological noise removal tools in both the cerebrum and the brainstem at 3 and 7 T; RETROICOR using external physiological recordings (hereafter RETROICOR‐EXT), and the ICA‐based methods FIX and PESTICA at 3 and 7 T.

## 2 MATERIALS AND METHODS

This section comprises descriptions of subjects, MR measurements and external physiological recordings (Sections 2.1 and 2.2), the PREPAIR algorithm for estimating physiological noise regressors (Section 2.3), data processing steps (Section 2.4), and comparison with other methods (Section 2.5).

### 2.1 Study participants

All healthy volunteers participating to this study were instructed to rest with eyes closed and to remain as still as possible. Ten subjects took part in the 3 T study (five females, age range 25–43 years old, mean age 31.1 ± 6.6 years old) and five subjects in the 7 T study (one female, four males, age range 26–38 years old, mean age 31.3 ± 4.6 years old).

### 2.2 Measurements

PREPAIR was tested on 3 T data with whole brain coverage and, to explore the dependence of the method on field strength and to analyze brain structures particularly prone to physiological noise, on 7 T data with a sagittal acquisition covering the brainstem. Protocols with a range of TRs and multiband (MB) factors were used in each study.

fMRI data were acquired using a multiband accelerated 2D‐EPI pulse sequence (Xu et al., 2013) developed at the Centre for Magnetic Resonance Research, University of Minnesota, Minneapolis U.S.A. (CMRR). The experiment comprised runs with four protocols with a range of TR, TE, flip angle, MB factor, number of slices (*N*), number of repetitions (NR), slice thickness, and GRAPPA acceleration factor detailed below and in Table 1.

**TABLE 1:** Acquisition parameters for the 3 and 7 T studies. First column indicates protocol names

	TR (ms)	TE (ms)	FA (°)	MB	*N*	NR	ST (mm)	GRAPPA
3T_TR_700	700	35.0	50	8	48	450	1.2	2
3T_TR_1020	1020	35.0	58	4	40	300	2.2	2
3T_TR_1520	1520	35.0	67	2	34	200	2.7	1
3T_TR_2000	2000	38.0	73	1	26	150	3.2	1
7T_TR_700	700	29.4	50	2	12	450	2.0	2
7T_TR_1020	1020	29.4	58	2	12	300	2.0	2
7T_TR_1520	1520	29.4	67	2	12	200	2.0	2
7T_TR_2000	2000	29.4	73	2	12	150	2.0	2

Abbreviations: FA, flip angle; MB, multiband acceleration factor; NR, number of repetitions; ST, slice thickness.

Each fMRI measurement was preceded by a low resolution, monopolar dual‐echo gradient‐echo prescan which was used to calculate the phase offset for each RF coil using ASPIRE (Eckstein et al., 2018). To generate channel‐combined EPI phase images, these phase offsets were subtracted from the phase of each channel of the EPI data for each time point, online in the image reconstruction environment, prior to calculating the angle of the magnitude‐weighted complex sum over channels (Bachrata et al., 2018).

#### 2.2.1 3 T study

Axial MRI data (excluding the brainstem) were acquired with a 3 T Siemens PRISMA (Siemens, Erlangen, Germany) scanner with a 64‐channel head coil. Neck coils were turned off. Slices were acquired in sequential ascending order, parallel to the AC–PC plane with a matrix size of 128 × 128, FOV = 210 mm × 210 mm (1.6 mm × 1.6 mm in‐plane resolution), distance factor of 20%, phase encoding direction: posterior–anterior, image bandwidth of 1565 Hz/px. Protocols parameters are summarized in Table 1.

#### 2.2.2 7 T study

Sagittal slices were acquired in sequential ascending order with a Siemens MAGNETOM 7T MRI Plus scanner with a single channel transit 32‐channel head coil Nova Medical coil (Wilmington, Massachusetts, USA) with a matrix size of 128 × 128, FOV = 220 mm × 220 mm (1.7 mm × 1.7 mm × 2.0 mm spatial resolution), distance factor of 30%, phase encoding direction: posterior–anterior, image bandwidth of 1445 Hz/px (see Table 1 for other protocol parameters and naming).

For comparison, physiological measurements of respiration and cardiac were made using a respiratory belt and an optical plethysmograph, respectively. PMU signals were recorded by the CMRR sequence at 1/400 s intervals, along with scanner trigger signals.

### 2.3 Estimation of the physiological regressors

**FIGURE 1:**
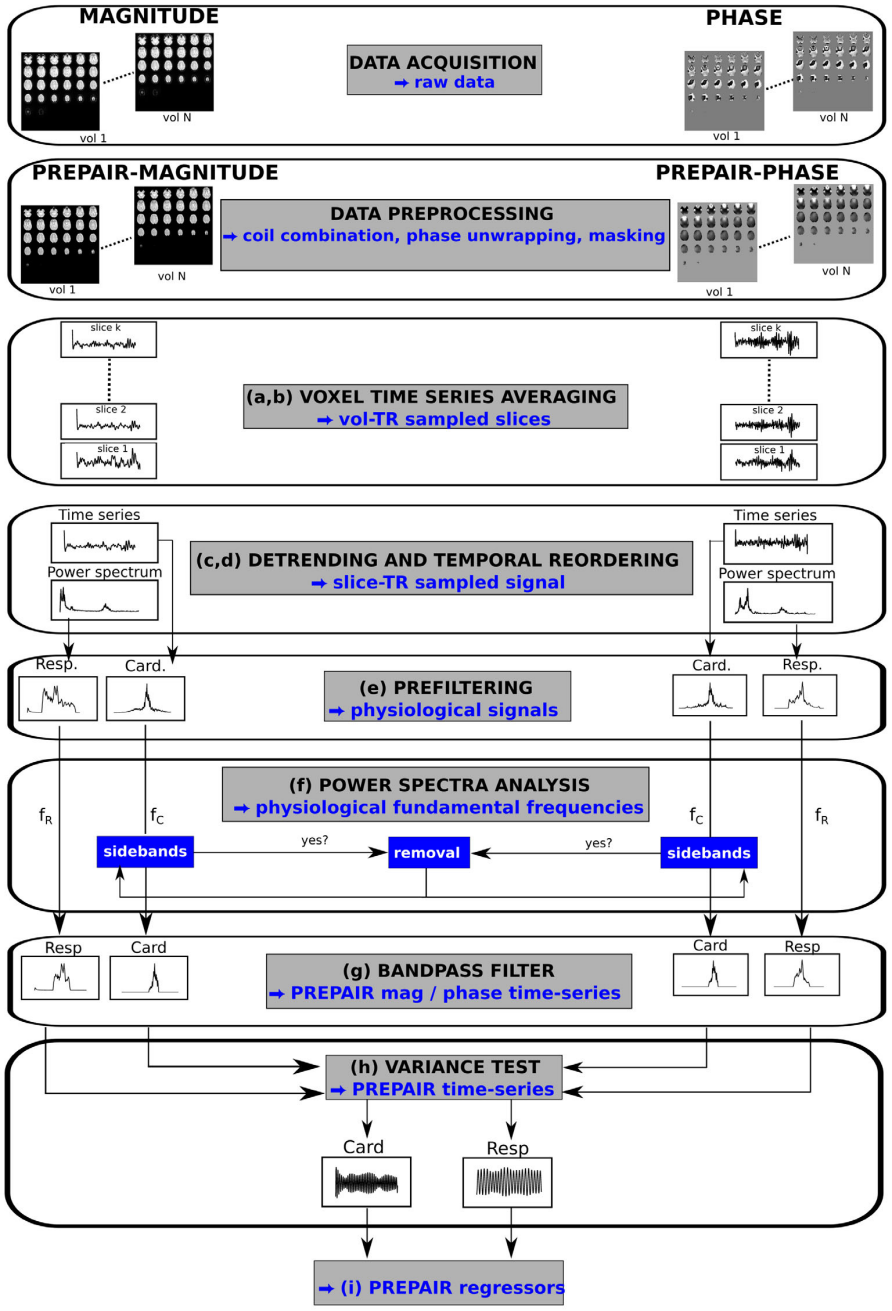
Steps in generating PREPAIR regressors (letters in brackets refer to steps described in Section 2.3). Raw magnitude and phase were preprocessed (for magnitude, with masking, for phase, with coil combination, unwrapping and masking). Magnitude and phase slices were averaged (a, b) then detrended and temporally reordered to yield slice TR sampled signals (c, d) from which respirators and cardiac time series were prefiltered (e). After deriving the corresponding physiological fundamental frequencies (f), the narrowed power spectra (g) underwent a variance test to choose between magnitude and phase physiological signals (h) to create the PREPAIR regressors (i)

The central aim of the PREPAIR approach is to generate two scalar signals sampled at ${\mathrm{TR}}_{\text{slice}}$ (rather than ${\mathrm{TR}}_{\mathrm{vol}}$)—one from the magnitude and one from the phase of the EPI data—and select from the two that which better describes respiration and cardiac fluctuations. These are used to generate physiological noise regressors for correction of the magnitude EPI data. The process consists of the following steps (illustrated in Figure 1, in which the letters below appear):

- Voxels with high SNR were identified using the criteria that their mean intensity over time in the magnitude was above 20% of the robust maximum defined as the 98th percentile of the values in the magnitude data over all voxels (Aslan et al., 2019).
- High SNR voxel values were averaged over each slice. For the phase, a weighted mean was used, in which weights were the corresponding magnitude values. This yielded a magnitude and phase time series for each slice, sampled at ${\mathrm{TR}}_{\mathrm{vol}}$.
- The dependence of the magnitude and phase values on imaging slice, which stems from different coverage and tissue composition, was eliminated by fitting and subtracting a third‐order polynomial to each slice time series.
- For phase and magnitude separately, signals derived from each slice were combined according to the acquisition order of each contributing slice. For SMS data, the signal in simultaneously acquired slices was averaged and the number of slices was reduced to NS = N/MB, with NS the reduced number of slices, where the reduction is by the number of simultaneously acquired slices, which is also equal to the number of RF excitations per TR. This resulted in a magnitude and phase time series with length NR × NS and sampling rate NS/TR.
- A “prefiltering” step, consisting of a band‐pass filter with typical ranges of [9–30] cpm (cycle per minute) for respiration and [42–96] bpm for cardiac, was applied to select physiological noise contributions to the magnitude and phase signals.
- The fundamental frequencies of the respiratory and cardiac noise, *f*_R_ and *f*_C_, respectively, were then identified with an iterative procedure in which the frequency with the highest power in the respective power spectrum was compared with signals with frequencies unrelated to physiological noise. Because band‐pass filters can be very aggressive, using filtering during this step would reduce or cancel out the power of signals of interests (stimulations, cardiac fundamental frequency, etc.) which can lie close to unrelated physiological signal frequencies. To prevent that, unrelated physiological noise frequencies were removed by setting their power to zero rather than applying a band‐pass filter to the time series.
- The respiratory and cardiac frequency spectra were then reduced in width by applying a band‐pass filter to the time series to retain only frequencies within a certain range around the fundamental frequencies.
- To determine whether the PREPAIR‐magnitude or PREPAIR‐phase physiological time series better represented physiological fluctuations in the data, the variance improvement after separately removing the respiratory and cardiac noise by linear regression was estimated and compared for both magnitude‐derived and phase‐derived signals. This led to the selection of either the PREPAIR‐magnitude or PREPAIR‐phase time series for respiratory‐related fluctuations, and the same for cardiac‐related fluctuations.
- Slice‐wise physiological noise regressors were modeled from the PREPAIR respiratory and cardiac time series as sine and cosine functions of the two first harmonics of the cardiac and respiratory cycle using the AFNI version of RETROICOR‐EXT.

### 2.4 Data processing

Phase wraps in raw phase images were removed by spatially unwrapping using ROMEO (Dymerska et al., 2021).

In the 3 T study, an image mask was generated using AFNI (Cox, 1996; Cox & Hyde, 1997), and applied to constrain analysis to the brain. In the 7 T study, no mask was used in the physiological noise identification or in the magnitude image correction, but analysis was constrained to a brainstem mask which included voxels in the medulla neighboring the spinal cord (which would be discarded with AFNI) by selecting voxels exceeding an intensity of 3000.

Data processing and analysis were performed using MATLAB 2018b (The MathWorks Inc., Natick, MA, USA).

### 2.5 Comparison with other methods

Among the existing physiological noise correction methods, we chose to compare PREPAIR with RETROICOR using external physiological recordings (RETROICOR‐EXT), PESTICA, which is ICA‐based but shares the similarity with PREPAIR that it also generates a slice TR signal, and FIX, another widely used ICA‐based tool.

For RETROICOR‐EXT, physiological signals recorded with the CMRR sequence were processed with the PhysIO Toolbox to model physiological noise regressors using RETROICOR with respect to the middle slice (Kasper et al., 2017).

PESTICA (Shin et al., 2016) (version 5.2) computes physiological estimators which can be filtered either via an interactive window inviting the user to zoom in to an appropriate region (supervised mode) or by using a default window of [48–85] bpm for cardiac and [10–24] cpm for respiration (unsupervised mode). Because of the large number of protocols and participants in our study, our analysis was performed in unsupervised mode, in which the frequencies of the respiratory and cardiac estimators ranged between [10–24] cpm and [45–85] bpm, respectively.

For FIX (version 1.06.15), MELODIC of FMRIB's Software Library (FSL version 4.15; www.fmrib.ox.ac.uk/fsl) was performed, with the number of components into which to separate the data set to 30. These were classified by FIX using the HCP7T_hp2000.Rdata training file. This was chosen because the spatial resolution was most similar to our spatial resolution. Due to the large number of tests performed in our study, we decided to run FIX in unsupervised mode, in which the threshold of good versus bad components was set to be the same for all subjects. This value was set to 50 after checking for a small number of subjects that resting state networks were not included in the list of components to be removed. This led to the identification of ~6 components/subject to for the 3 T study and approximately two components/subject for the 7 T study to be filtered out.

#### 2.5.1 Correlation with the physiological recordings

Because the PREPAIR physiological time series were band‐pass‐filtered such that the window of their power spectra was centered on their corresponding fundamental frequency with a range of frequencies around it (as described in Section 2.3, step g), we applied the same band‐pass filter to the respiratory and cardiac waveforms derived from the different methods to narrow their power spectrum to the same window size as PREPAIR's, to ensure a fair comparison. All correlations in Section 3.1 accounted for lags between the physiological noise from the fMRI data and the physiological recordings by measuring the cross correlation between those time series. Although FIX provides the list of components removed from the fMRI data, no correlations were computed, as that would have required a manual inspection of the classified components to only include those which were physiological noise‐related; a process which is both time consuming and subjective.

#### 2.5.2 Spectral analysis

To facilitate the visualization of the physiological noise present in 4D time series of the corrected magnitude images in Section 3.2, data were converted into a single time series sampled at the slice TR by first averaging over the voxels in each slice, then reordering those time series according to the slice acquisition times. We then performed a spectral analysis of these time series using the Chronux toolbox (Bokil et al., 2010) to produce spectra of frequencies of signals varying in average sliding windows of 45 s and a step‐size of 4 s (Supplementary Information, Figure S2 [the first five subjects of the 3 T study], Figure S3 [the last five subjects of the 3 T study], and Figure S4 [7 T study]).

To assess the image correction efficiency of each method presented in Tables 3 and 4, we calculated the proportion of noise removed in the respiratory and cardiac bands (including the second harmonic when present) of the uncorrected magnitude data by computing the relative change in power for each voxel between the uncorrected and corrected magnitude, and then by averaging over each slice. Negative values of the proportion mean the correction added noise, zero values mean that no noise was removed, and positive values mean that the correction was effective (a value of one indicates that the level of physiological noise decreased by 100%). To determine which method preserved the integrity of the signal outside the respiration and cardiac areas, we also included the proportion of power fluctuation, that is, the change in the power signal (increase or decrease) after correction. Values close to zero would indicate that a method was highly effective in preserving the original signal.

#### 2.5.3 tSNR improvement in anatomical regions

To assess tSNR improvement in regions of the brain typically affected by physiological fluctuations, we performed a statistical analysis in manually drawn 3D ROIs in the visual and insular cortices in the 3 T study (subjects 1, 2, 4, 7, and 10), and the brainstem in the 7 T study.

## 3 RESULTS

In this section, we illustrate the physiological signals generated with PREPAIR and compare physiological noise correction between PREPAIR, RETROICOR‐EXT, PESTICA and FIX. In Section 3.1, the quality of physiological waveforms obtained with each method is compared with that from the external signals. The efficacy of the noise removal with each method is shown in Section 3.2 and its effectiveness in the improvement of the magnitude image in Section 3.3.

Because all intermediate data needed for this assessment are not available with FIX (e.g., slice TR‐sampled physiological regressors), the comparison between PREPAIR and FIX was limited to the analysis of the corrected magnitude image (Section 3.2).

### 3.1 Physiological noise identification

#### 3.1.1 Illustration of the PREPAIR algorithm

**FIGURE 2:**
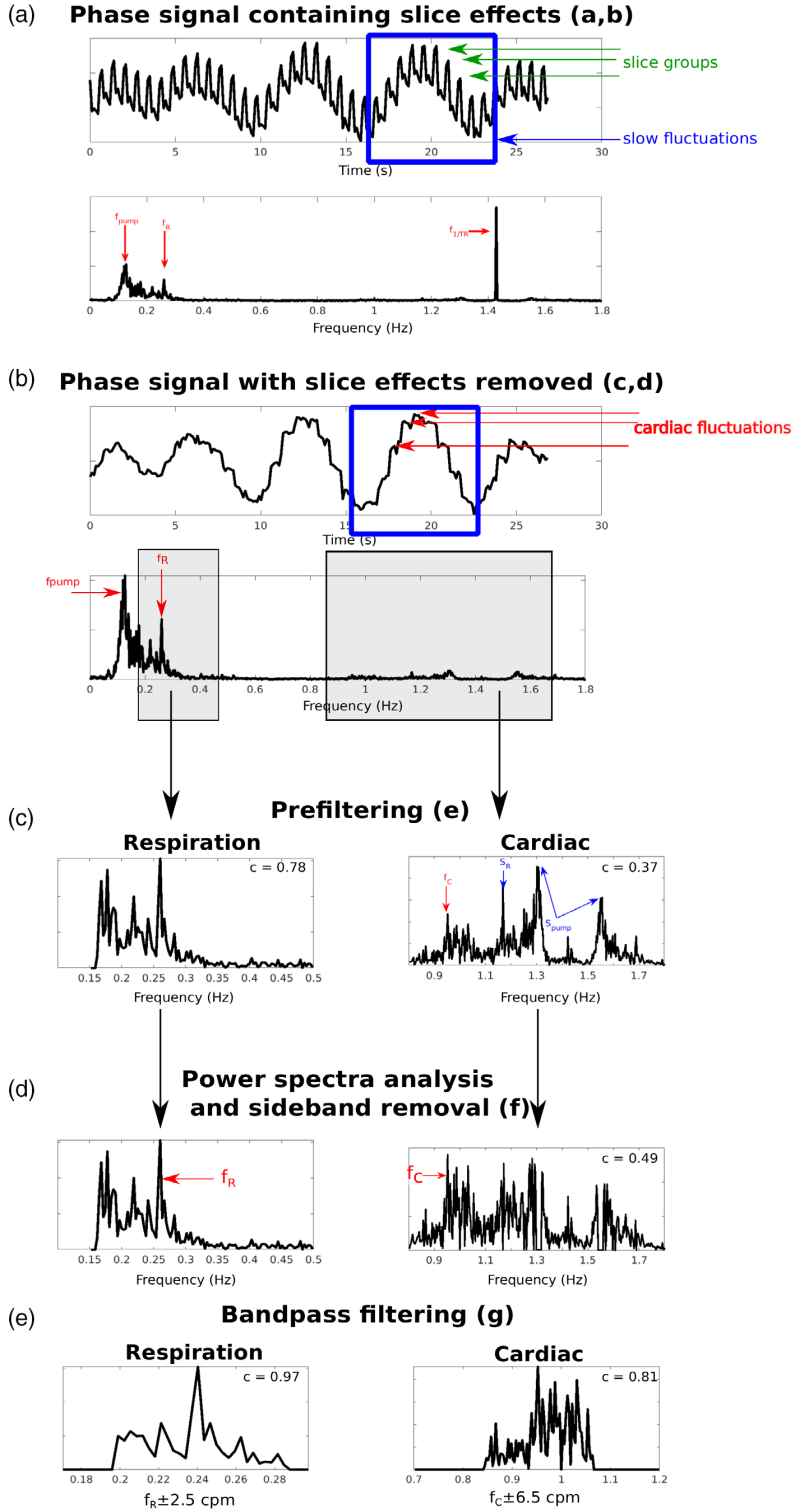
Illustration of physiological noise identification in the slice TR sampled phase signal of subject S6, 3T_TR_700 (a), which is dominated by periodic slice effects (green arrows) sampled at *f*_1/TR_, and slow fluctuations (blue square) comprising respiration (sampled at 1/fR) and cold head pump (sampled at 1/fpump). After removing the slice effects (b), physiological fluctuations were prefiltered from the phase signal (c) and unrelated physiological frequencies (sidebands SR and Spump) were removed to find the correct cardiac fundamental frequency *f*_C_ (d). Respiratory and cardiac signals were band‐pass filtered around *f*_R_ and *f*_C_, respectively. Correlations improvement with the external signals are indicated for each step of the algorithm

The effect of the steps detailed in Section 2.3 for physiological noise identification is illustrated in Figure 2 with the phase data of one subject (S6, 3T_TR_700). Panel (a) shows the slice TR sampled phase signal (top). Before removing slice effects, both slow fluctuations (blue square) and faster fluctuations (slice groups, green arrows) are apparent. The Fourier spectrum (bottom) is dominated by three peaks: *f*_pump_ at 0.13 Hz, a disturbance related to the cold‐head helium pump affecting the flow of the gradient cooling water (as described in Hagberg et al. (2012)), *f*_R_, the respiratory fundamental at 0.25 Hz and *f*_1/TR_ at ~1.43 Hz caused by slice reordering. Slice effects were removed by detrending (Section 2.3, c and d) (panel (b)), reducing the power of *f*_1/TR_ and revealing rapid variations of cardiac origin (red arrows). In panel (c), respiratory fluctuations (left) were band‐pass filtered from the power spectrum (Section 2.3, e), resulting in a correlation of 0.78 with the external signal for the case illustrated. The cardiac fluctuations (right) extracted from the time series by applying a second‐order Butterworth band‐pass filter had a correlation value of 0.37 with the external signal. This Butterworth filter includes frequencies beyond the chosen interval so that unusual cardiac rhythms can be identified. In this cardiac power spectrum, we observe multiple peaks: the cardiac fundamental *f*_C_ and sidebands caused by *f*_pump_ located at *S*_pump_ = *f*_1/TR_ ± *f*_pump_ and by *f*_R_ located at *S*_R_ = *f*_1/TR_ ± *f*_R_, respectively (the upper sideband does not appear as it lies outside the cardiac range). Here, *f*_C_ was well below *S*_pump_ and *S*_R_ which would be wrongly identified as the fundamental. After filtering out all sidebands (Section 2.3, f), *f*_C_ was correctly identified (panel (d)) which improved the correlation with the external signals to 0.49. In panel (e), the intervals of the respiratory and cardiac frequencies were reduced to ranges around *f*_R_ and *f*_C_ (Section 2.3, g). These ranges were established by computing the average standard deviation of the respiration and cardiac rates in the signals obtained from the external signals over all subjects, protocols and field strengths, which yielded 2.5 cpm and 6.5 bpm for respiration and cardiac, respectively. Frequencies outside the ranges *f*_R_ ± 2.5 cpm and *f*_C_ ± 6.5 bpm were eliminated with a band‐pass filter, leading to correlation for respiration and cardiac with the external signals of 0.97 and 0.81, respectively.

#### 3.1.2 Comparison of the physiological waveforms

Table 2 lists the mean correlation over subjects of PREPAIR‐magnitude and PREPAIR‐phase waveforms with the external signals and the proportions of cases in which the PREPAIR‐magnitude and PREPAIR‐phase regressors were selected for respiratory and cardiac noise. For the 3 T study, PREPAIR‐phase matched extremely well the external signals and modeled respiration fluctuations better than PREPAIR‐magnitude for all subjects and protocols. For cardiac, correlation values with PREPAIR‐phase were lower, and were identified as the best regressor in 40% of cases for 3T_TR_700 and 3T_TR_1020 and 60% for 3T_TR _1520 and 3T_TR_2000 protocols. For the 7 T study, PREPAIR‐phase also provided a better description of respiration‐related fluctuations for all subjects and protocols. For cardiac, the PREPAIR‐magnitude waveform was used to build the cardiac regressors in all subjects and protocols.

**FIGURE 3:**
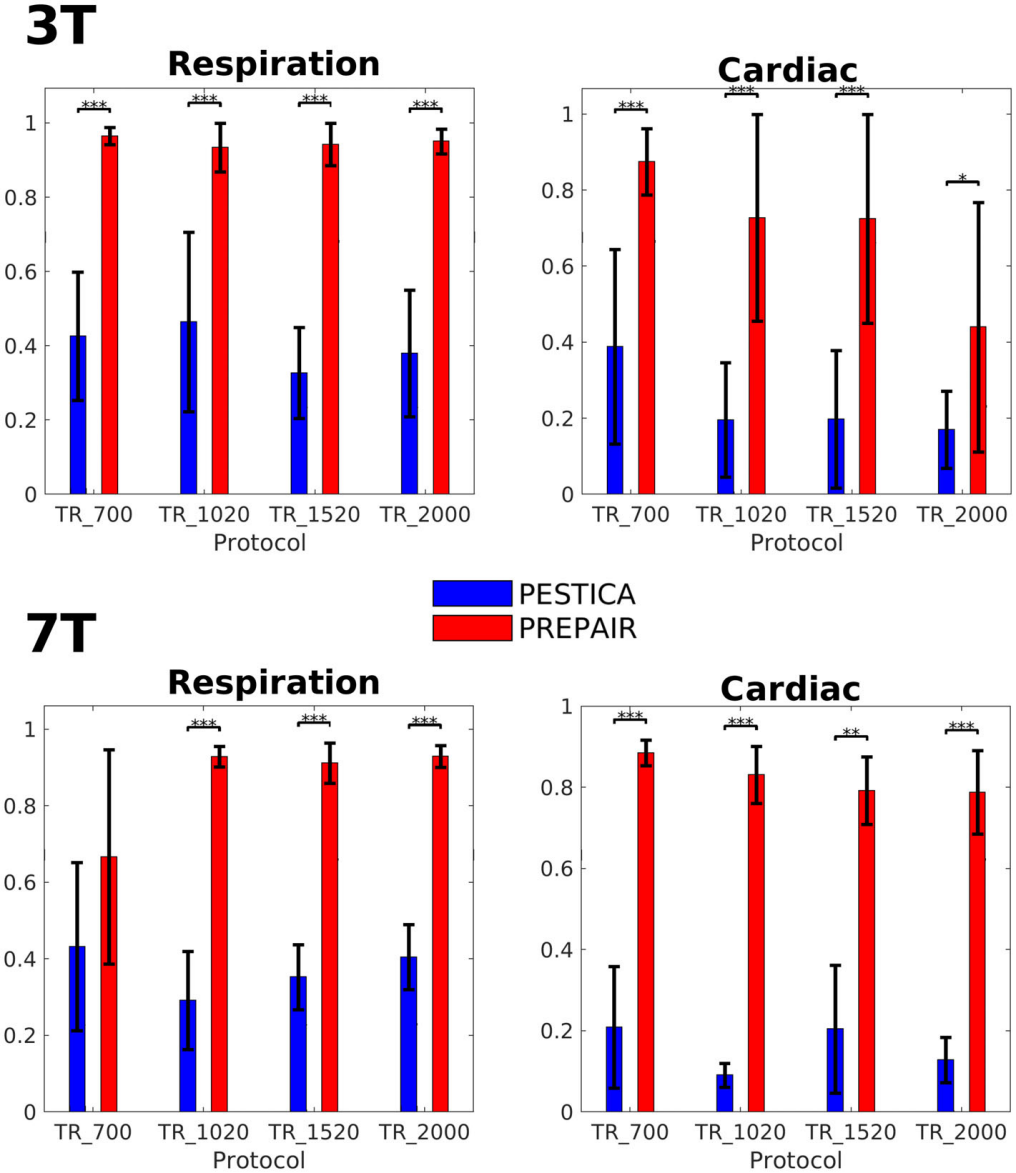
Correlation of PESTICA (blue) and PREPAIR (red) with the externals signals across subjects for respiration and cardiac for the 3 T study (top) and 7 T study (bottom). Most mean correlations of PREPAIR are significantly (*p < .05; **p < .01; ***p < .001) above those of PESTICA

Given the important contribution of the phase and magnitude data in the process of noise identification, we compared, in Figure 3, the correlation of the PREPAIR respiratory and cardiac waveforms selected by the algorithm with that of PESTICA (all PESTICA estimators are presented in Supplementary Information, Figures S8 and S9). For the 3 and 7 T studies, PREPAIR performed significantly better (*p < .05; **p < .01; ***p < .001) than PESTICA except for 7T_TR_700 for respiration.

The results of this section demonstrate that PREPAIR can accurately detect respiratory and cardiac fluctuations in the fMRI data. The respiratory and cardiac waveforms selected by the algorithm were used to produce physiological noise regressors for image correction.

### 3.2 Physiological noise removal

#### 3.2.1 Comparison of uncorrected versus corrected power spectra

**FIGURE 4:**
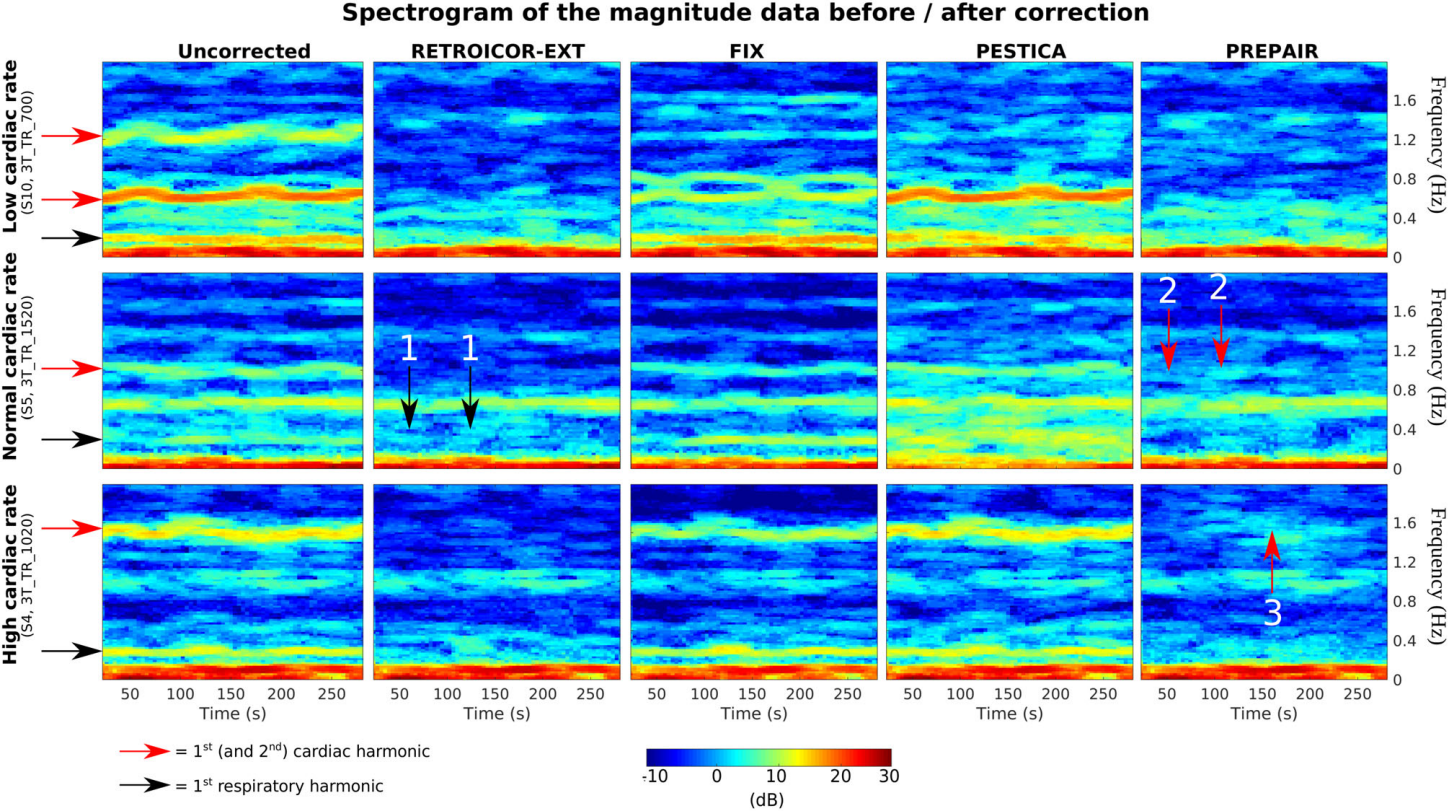
Spectrograms of uncorrected (left) versus corrected magnitude data. Frequencies (y axis) were truncated at 1.7 Hz and rows were scaled to the same power expressed in decibels (color scale). Horizontal black and red arrows indicate the first (and second when applicable) harmonic of the respiratory and cardiac noise, respectively, obtained with the external signals. PREPAIR showed effective noise removal in comparison with the other methods, even with unusual cardiac rates (top and bottom). Numbered vertical arrows show islets of remaining power after noise correction

The effectiveness of noise removal with the methods under consideration was investigated by performing a spectral analysis of the magnitude data prior to and following correction. In the analysis below, we compare the power reduction of physiological noise bands with the three methods for three selected subjects in the 3 T study who had low (top: S10, 3T_TR_700), normal (middle: S5, 3T_TR _1520) and high (bottom: S4, 3T_TR_1020) cardiac heart rate. In Figure 4, the variation of the respiratory and cardiac fluctuations over time obtained with the external recordings is indicated with a black and red arrows (two if the second harmonic is visible) in the uncorrected magnitude image (left panel), respectively. Spectrograms were scaled to the same minimum and maximum power expressed in decibels (color scale).

- In the top panel, showing results for a subject with a slow pulse, the respiratory noise was close to 0.3 Hz and the cardiac first and second harmonics were at ~0.6 and 1.2 Hz. The respiratory removal was as effective with PREPAIR and RETROICOR‐EXT, while in PESTICA, the correction was partial, and in FIX noneffective. The contributions of the first and second cardiac harmonics were completely removed by correction with PREPAIR and RETROICOR‐EXT while PESTICA and FIX only removed the second harmonic.
- In the middle panel, showing results for a subject with a typical pulse, the respiratory noise lies near 0.3 Hz and the cardiac first harmonic around 1.0 Hz. Concerning the respiration, PREPAIR slightly outperformed RETROICOR‐EXT, for which some islets of power remain in the spectrograms (black arrows with label 1), and PESTICA and FIX added noise. As for the cardiac noise, remaining power is visible in the spectrogram of PREPAIR compared to RETROICOR (red arrows with label 2), but neither FIX nor PESTICA filtered this noise out.
- In the bottom panel, showing results for a subject with a fast pulse, the respiratory noise peak was around 0.3 Hz and the cardiac first harmonic around 1.6 Hz. Physiological noise removal was only successful with RETROICOR‐EXT and PREPAIR, albeit with one islet of residual power (red arrow with label 3) in PREPAIR's spectrogram.

**FIGURE 5:**
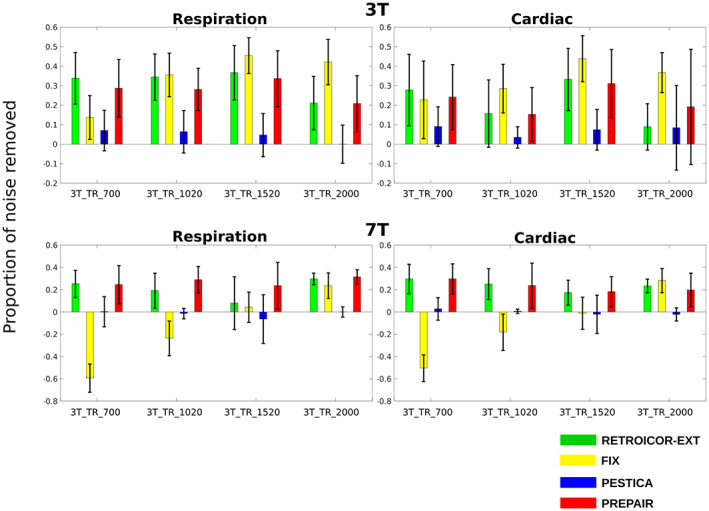
Proportion of respiratory and cardiac noise removed by each method and for each protocol across subjects. Physiological noise correction performed with PREPAIR was as effective as that of RETROICOR‐EXT but more effective than FIX at 7 T only and PESTICA at both 3 and 7 T

To estimate the overall performance of each method after physiological noise correction, we computed the mean proportion of physiological noise removed and of fluctuation outside the physiological noise regions. Figure 5 shows the mean proportion of physiological noise removed in the 3 T (top panel) and 7 T (bottom panel) studies across subjects. For the 3 T study, PREPAIR gave a correction which was as effective as RETROICOR‐EXT, slightly less effective with FIX for some protocols, but more effective than PESTICA. For the 7 T study, PREPAIR performed better than PESTICA and FIX for all protocols, and similarly to RETROICOR‐EXT. Table 3 compiles the mean and standard deviation (in percentage) of the total noise removed by each method over all subjects and protocols for the 3 T study. For respiration (first row), PREPAIR (30.0%) was as effective as RETROICOR‐EXT (32.1%) and slightly less than FIX (33.7%), with the performance of PESTICA (4.8%) being significantly lower (*p* < .001). For cardiac (second row), PREPAIR (20.0%) performed similarly to RETROICOR‐EXT (20.7%), significantly better than PESTICA (6.0%: *p* < .001). However, FIX (33.7) removed significantly more cardiac noise than PREPAIR (*p* < .001). Table 4 shows similar results for the 7 T study. For respiration, PREPAIR (27.3%) was as effective as RETROICOR‐EXT (28.7%), and removed significantly more respiratory signals than FIX (−17.1%: *p* < .001) and PESTICA (−2.0%: *p* < .001). For cardiac, PREPAIR (22.3%) also performed significantly better than FIX (−10.1%: *p* < .001) and PESTICA (0.0%: *p* < .001), and similarly to RETROICOR‐EXT (23.6%).

A reliable physiological noise correction method should be effective in removing the narrowband physiological noise and leave signal outside these regions unchanged. To assess the extent to which all methods modified signal power outside the physiological bands, we estimated the proportion of power fluctuation outside physiological noise regions. For the 3 T study (last row of Table 3), PREPAIR only altered the magnitude signal by 1.9%, comparable to RETROICOR‐EXT (1.4%), which was significantly less than FIX (28.4%, *p* < .001) and PESTICA (3.3%, *p* < .01). For the 7 T study (last row of Table 4), PREPAIR (2.5%), RETROICOR‐EXT (1.1%), and PESTICA (1.5%) all reduced the power fluctuation to similar level, whereas the change of power outside the physiological bands was significantly larger with FIX (−27.8%: *p* < .001).

This section demonstrates that PREPAIR effectively removed physiological fluctuations from magnitude time series data while preserving signal of interests, which is crucial for the detection of task‐driven voxels.

### 3.3 tSNR improvement in anatomical regions

**FIGURE 6:**
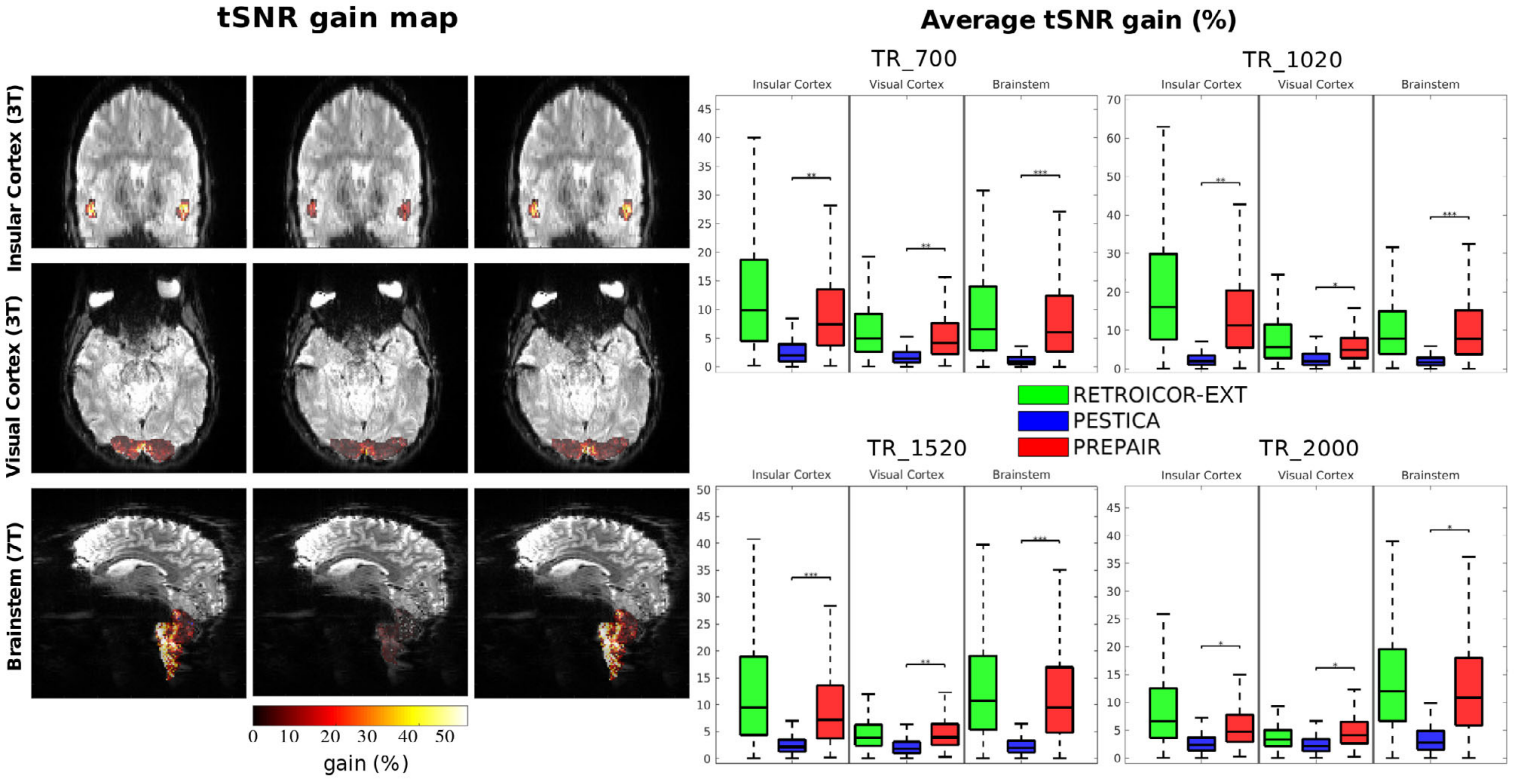
Temporal signal‐to‐noise‐ratio (tSNR) gain map (S2, 3T_TR_700) in different anatomical regions (left panel). The level of improvement with PREPAIR was comparable with RETROICOR‐EXT and larger than with PESTICA. For all protocols and anatomical regions (right panel), tSNR gain with PREPAIR was significantly higher than PESTICA (*p < .05; **p < .01; ***p < .001). The level of tSNR improvement with PREPAIR in the insular and visual cortices is slightly weaker than RETROICOR‐EXT but comparable in the brainstem

tSNR increases in the insular cortex, visual cortex and brainstem for S2, TR_700 are illustrated in the left panel of Figure 6. The improvement in tSNR was similar with RETROICOR‐EXT and PREPAIR, and larger than PESTICA, particularly in the brainstem. In the assessment over all subjects, shown in the boxplots in the right part of the same figure, it is apparent that the mean tSNR increase with PESTICA was less than 5% for all protocols, with significant differences between PESTICA and PREPAIR in all protocols and anatomical regions. The mean for RETROICOR‐EXT and PREPAIR was above 5% in all cases. In the 3 T study, RETROICOR‐EXT was slightly more effective than PREPAIR in the insular cortex (by a few percent) for all protocols. In the visual cortex, tSNR gain with PREPAIR was slightly lower for 3T_TR_700 and 3T_TR_1020, comparable to RETROICOR‐EXT for 3T_TR_1520 and slightly better than RETROICOR‐EXT for 3T_TR _2000. In the brainstem, 50th percentiles of PREPAIR were always similar to those of RETROICOR‐EXT for all protocols, although 75th percentiles of RETROICOR‐EXT for 7T_TR_700, 7T_TR_1520, and 7T_2000 were slightly higher.

We excluded FIX from this comparison, as we found a wide spread of standard deviations in the mean variance improvement across subjects (Supplementary Information, Figure S5), suggesting that other sources of noise and signal variation have been removed. Thus, tSNR improvements through FIX may not stem from the removal of the physiological noise, but from other signal and noise components. This was verified by identifying the FIX components that were not physiological‐related, that is, when one of the main three peaks of the power spectra did not correspond to the physiological fundamental frequency (or was aliased) with a tolerance of 10% around it. Over all protocols and subjects of the 3 T study, we found that 35% of the components selected by FIX were not physiological noise‐related but related either to eye motion (Supplementary Information, Figure S6) or to a resting state network (Supplementary Information, Figure S7).

## 4 DISCUSSION

We have developed a physiological data estimation and correction method called PREPAIR, which identifies physiological fluctuations from fMRI data without the need for supplementary external physiological recordings. The novel aspects of PREPAIR are that it uses both magnitude and phase information, physiological noise is sampled at the slice TR rather than the volume TR—so high frequency cardiac fluctuations are critically sampled—and it does not require user interaction to generate the magnitude or phase regressors or select which of the two best captures respiratory and cardiac fluctuations.

We attribute the utility of the phase in identifying respiration‐related signals as being due to the sensitivity of the phase to change in the Δ*B*_0_ field where there is motion of structures with large susceptibility differences; primarily in this case the chest (Raj et al., 2001). Cardiac changes were generally better identified with the magnitude—50% of the subjects and protocols at 3 T and 95% at 7 T (see Table 2)—because of the large changes to the magnitude generated by spin history effects. In a few cases where the correlation between the phase and the external recordings was much higher than with the magnitude, PREPAIR would have failed if only the magnitude data had been used (see Supplementary Information, Figure S1). Besides, physiological signals can have different shape, behavior, and offsets compared to signal changes we extract from images. Therefore, they will never perfectly match. When the signals do not match perfectly, less variance related to physiological will be removed compared to signals which match better, as illustrated in Figure B‐11 (Supplementary Information).

**TABLE 2:** PREPAIR‐phase and ‐magnitude correlation with the external signals, and proportion of PREPAIR‐phase and ‐magnitude (phase vs. magnitude) selected by the algorithm for deriving the PREPAIR regressors. PREPAIR‐phase modeled respiratory fluctuations better than PREPAIR‐magnitude for both 3 and 7 T studies and all PREPAIR‐phase signals were used for deriving respiratory regressors. For cardiac, PREPAIR‐phase and ‐magnitude contributed equally for the 3 T study over all protocols, whereas for the 7 T study, PREPAIR‐magnitude was chosen in almost all protocols (except for 7T_TR_1020)

3 T		3T_TR _700	3T_TR _1020	3T_TR _1520	3T_TR _2000
Respiration	Correlation: phase	0.97 ± 0.02	0.97 ± 0.03	0.96 ± 0.04	0.95 ± 0.03
	Correlation: magnitude	0.66 ± 0.21	0.60 ± 0.19	0.63 ± 0.25	0.18 ± 0.28
	Phase vs. magnitude	100%:0%	100%:0%	100%:0%	100%:0%
Cardiac	Correlation: phase	0.72 ± 0.28	0.61 ± 0.36	0.73 ± 0.25	0.43 ± 0.28
	Correlation: magnitude	0.90 ± 0.10	0.75 ± 0.23	0.68 ± 0.24	0.25 ± 0.24
	Phase vs. magnitude	40%:60%	40%:60%	60%:40%	60%:40%

7 T		7T_TR_ 700	7T_TR_1020	7T_TR_1520	7T_TR_2000
Respiration	Correlation: phase	0.76 ± 0.24	0.93 ± 0.02	0.93 ± 0.04	0.93 ± 0.02
	Correlation: magnitude	0.75 ± 0.34	0.78 ± 0.22	0.59 ± 0.31	0.33 ± 0.44
	Phase vs. magnitude	100%:0%	100%:0%	100%:0%	100%:0%
Cardiac	Correlation: phase	0.79 ± 0.11	0.67 ± 0.33	0.70 ± 0.18	0.59 ± 0.32
	Correlation: magnitude	0.89 ± 0.03	0.85 ± 0.06	0.80 ± 0.09	0.80 ± 0.11
	Phase vs. magnitude	0:100%	20%:80%	0%:100%	0%:100%

Reliance on phase data could be viewed as a limitation of the PREPAIR approach, as phase images are not currently routinely reconstructed and stored in fMRI experiments. The use of phase requires a solution for the phase‐sensitive combination of RF coil signals, but also phase unwrapping and the removal of unwanted signals from sources such as the cold‐head helium pump which affect the phase signal to a larger extent than the magnitude fMRI signal (Hagberg et al., 2012). However, increasing interest in methods which use the phase for dynamic distortion correction (Dymerska et al., 2018), QSM (Sun & Wilman, 2015), and functional QSM (Balla et al., 2014) have led to the development of effective solutions to these problems.

Considering coil combination, MRI scanner vendors have effective coil combination methods for systems with a body coil (e.g., SENSE (Pruessmann et al., 1999) and prescan normalize combined with adaptive combination (Jellus & Kanengiesser, 2014)). For 7 T systems, a multi‐echo ASPIRE prescan can be used to calculate phase offsets needed to combine separate channel EPI data (Eckstein et al., 2018) as was done here, with alternatives being offered by the CMRR sequence (Xu et al., 2013), which uses SENSE, and implementations of the VRC approach (Parker et al., 2014), to give a few examples.

For phase unwrapping, recent faster methods (Dymerska et al., 2021; Karsa & Shmueli, 2019) mean that this step can be performed sufficiently quickly and reliably that no longer poses an obstacle to phase imaging with EPI.

In regards to signals which disrupt the phase, the main frequency generated by the pump lies near the respiratory fundamental frequency for TR = 700 ms but without overlap, as seen in Figure 2. Our results have revealed that this perturbation would move away from the frequency range occupied by respiratory fluctuations with increasing TR, which agrees with Figure 1 in Hagberg et al. (2012) where the main cold pump peak lies at 0.67 Hz for a faster TR (180 ms), between typical respiration and cardiac frequency ranges. Protocols with TR below 700 ms would move this major perturbation to higher frequencies, which could limit the detection of the respiration fundamental frequency in the phase time series. Furthermore, the presence of sidebands can make it difficult to identify the fundamental physiological frequencies. Sidebands occur when a carrier signal is modulated by a lower frequency with high power, producing mirrored signals around the carrier. For instance, the coupling of the respiratory and the cardiac cycles—caused by the respiratory modulation of arterial‐induced pulsations—leads to frequency contributions at the sum of and the difference between the fundamental cardiac frequency (the carrier) (Brooks et al., 2008; Raitamaa et al., 2021). The reordering process can also engender sidebands at *f*_n/TR_ ± *f*_m_ due to the amplitude modulation of the carrier signal *f*_n/TR_ (i.e., 1/TR and its harmonics) by a lower frequency *f*_m_, which can either be respiratory and helium cold pump frequencies in the case of phase data, or scanner drifts in the magnitude data. Those *f*_m_ frequencies are known to be dominant in fMRI data. This step can be critical for long TRs if the width of *f*_m_ is large (which is the case for respiratory signals but not for scanner drifts) as the resulting sidebands can lie much lower than typical cardiac frequencies and reach the respiratory domain. Because these only affect the phase signal, the magnitude time series will enable PREPAIR to overcome these challenges in either situation in case the phase fails to capture physiological signals. Indeed, for fast and low TRs, the magnitude component of PREPAIR proved to be able to detect respiratory and cardiac fluctuations with a very good agreement with the external recordings acquired at both 3 and 7 T (see Table 2).

Generally, the bandwidth in EPI is chosen to be as low as possible while still allowing the TE to be reached, with the avoidance of forbidden frequencies and minimization of ghosting being other considerations. Here, we selected the bandwidth according to these criteria, rather than selecting the bandwidth due to any influence it would have on the phase. Other than the generally behavior that the bandwidth affects the SNR (inversely proportional to the square root) it only affects eddy currents, generating, in the phase, a (quasi)‐linear gradient in the readout direction, which is the same for all volumes, so it does not affect the time series.

A potential important advance in the use of phase data that is brought out in Table 2 for the 3 T study. Correlation of the PREPAIR‐magnitude to external signals seems to decrease with longer TRs, when they remain almost constant with PREPAIR‐phase. This would suggest that the coupling of physiological noise in BOLD data is much stronger in less undersampled magnitude data whereas the phase signal coupling is not affected by the number of samples. A preliminary conclusion would be that phase data provide more consistent and reliable physiological fluctuations than the magnitude data especially in undersampled data. This would explain the higher proportion of PREPAIR‐phase waveforms selected by the algorithm for TR greater than 1520 ms (see Table C‐3 in Supplementary Information).

Regarding the 7 T study limited to the brainstem, we chose an arbitrary value of 3000 based on our image intensity to include part of the brainstem (including part of the spinal cord in our FOV) which would have been excluded with classical masking methods such as AFNI. This threshold could be generalized to a certain percentile (here 75th percentile), however studies specifically dedicated to the brainstem would certainly benefit from more robust masks, for example, the probabilistic mask from the Harvard‐Oxford brain atlas (Desikan et al., 2006; Frazier et al., 2005; Goldstein et al., 2007; Makris et al., 2006).

The slice signal detrending used slice TR sampling of physiological noise, but some volume TR dependency remains, and PREPAIR could fail to detect cardiac fundamental frequencies when they are too close to 1/TR frequency or harmonics thereof, especially for TR = 1020 ms (1/TR = 0.98 Hz) with subjects having a cardiac heart rate near 1 Hz, which is quite typical, or include the remains of this perturbation in the physiological waveforms. This could explain the poor correlation with the external physiological signals for TR_2000 (see Figure 3). Despite that, PREPAIR never significantly performed less well than RETROICOR‐EXT in tSNR improvement and even performed comparably to RETROICOR‐EXT in the brainstem (see Figure 6).

The default option in the PhysIO Toolbox is to downsample regressors to a reference slice used in a single GLM applied to the whole 3D brain as in SPM (the PhysIO toolbox is integrated in SPM in default mode). Alternatively, slice‐specific regressors can be computed by generating as many text files as the number of slices as mentioned in Kasper et al. (2017). We decided to apply the PhysIO toolbox as it is implemented and used in current practice by users of SPM or FSL and compare the PREPAIR regression to that of RETROICOR‐EXT from physiological noise regressors obtained from the default mode of the PhysIO toolbox. However, the use of slice‐wise regressors is highly recommended as they might improve the performance of the regression in targeted structures of the brain like the brainstem as in Harvey et al. (2008).

Our model did not include physiological noise originating in fluctuations in the heart rate (Akselrod et al., 1981; Cohen & Taylor, 2002; Otzenberger et al., 1998) and subtle changes in the depth and rate of breathing (Wise et al., 2004), which occur at much lower frequencies (<0.1 Hz) than that of the cardiac cycle (~1 Hz) and respiration (~0.3 Hz). Including these slower fluctuations as regressors in the analysis can improve the statistical power of fMRI studies and the detection of task‐related BOLD signal changes (Birn et al., 2006; Chang et al., 2009; Shmueli et al., 2007); something which could be added to the PREPAIR output without any fundamental changes to the processing.

In our experiment, no special instructions were given to the participants on how to breathe, so the breathing here was a natural breathing, typical of that which would be encountered in other fMRI experiments. We noticed that the widespread range of frequencies (±2.5 cpm around the respiratory frequency fundamental) in the respiration power spectrum reflect the irregular amplitude of the chest motion during breathing. However, if a breathing task requires too extreme breathing conditions (deep breathing and fast breathing for instance) PREPAIR might fail because the two main peaks would be too far from each other to be contained in the ±2.5 cpm frequency range around the main peak. If the breathing task is such that the two peaks are not too far from each other, PREPAIR will remove them from the magnitude data.

Cardiac and to a lesser extent respiratory effects on image intensity will not be synchronized across the imaged volume. For instance, cardiac‐driven signals will have phase offsets from one slice location that is dependent on the relative distances from the heart and the position of the measured signals through the cardiac cycle. Estimated blood velocities in the Circle of Willis (Ahn et al., 1991) are generally between 11.7 cm/s (ophthalmic artery) and 58.5 cm/s (middle cerebral artery), which could create a time offset of about 100–500 ms for an imaging slab of 12 cm and MB = 2. That delay could, potentially, reduce the accuracy of estimates generated from slice‐averaged signals. Thus, one can question if deriving cardiac estimates from slice‐averaged signals (as in our method) might reduce sensitivity, since simultaneously sampled cardiac processes will be at different positions in the cardiac cycle. This was checked by calculating, for all subjects and protocols, the proportion of cardiac and respiratory noise removed with regressors obtained without averaging slices and compared that with the proportion of cardiac and respiratory noise removed with regressors obtained without averaging slices (our approach). There was a small lag (of between 70 and 150 ms) between the averaged slices (see Supplementary Information, Figure A‐10 and Table A‐1). This did not impact the image correction as we found that proportions of respiratory and noise removed without slice‐averaging were statistically similar to values with averaged slices. We attribute the slightly higher performance of our original approach (with slice averaging) to the fact that the influence of the edge slices, which are susceptible to motion, is reduced when averaged with simultaneously acquired slices at the center of the brain, making the contribution of physiological signals more important in the time series.

An important criterion for selecting a physiological noise correction method for multisubject studies is whether the approach can be executed without user intervention/unsupervised. FIX is challenging to use in a study with a large number of subjects. First, it is very time consuming, because MELODIC‐ICA must be run prior to classification. Second, the appropriate training data set used for the classification should be carefully chosen as it depends on the scanner, sequence type, set of subjects, voxel size, and TR. We ran FIX with a training dataset corresponding to our in‐plane resolution (1.6 mm × 1.6 mm), 7 T scanner, and sequence type (CMRR as used in the HCP). Also, it is recommended to train classifiers on our own data sets prior to classification, then extend to a larger sample, which is also time consuming and would require scanning more subjects. Thirdly, FIX is prone to include components of interest (i.e., signal changes related to activation) because of the dependence on the threshold parameter (see Supplementary Information, Figure S5), which controls the ratio of good to bad components, and which is subject dependent, as seen from the number of components labeled as physiological noise (see Section 2.5). This would explain the low performance of FIX at 7 T compared to 3 T. Finally, but importantly, an optimal noise removal procedure with FIX requires manual checks that activation‐related components are not excluded, which can be onerous. PESTICA can be run in either unsupervised or supervised mode. Running it in supervised mode involves defining the range of frequencies suspected to be physiological noise‐related and may have improved its performance. The performance which could be achieved was assessed by inspecting all the power spectra of respiratory and cardiac estimators generated by PESTICA software (Supplementary Information, Figures S8 and S9; delta values are for the dispersion of the fundamental physiological frequencies with respect to those from the external recordings). Among these, narrowing the frequency distribution would have been challenging for 48 power spectra (60%) because either there was no obvious distribution (for instance, Cardiac: S5_3T_TR_700, S9_3T_TR700; Respiration: S4_3T_TR_2000, S9_3T_TR_1520), or there were multiple distributions (for instance, Cardiac: S2_3T_TR_1520, S2_3T_TR_2000; Respiration: S9_3T_TR_1020, S10_3T_TR_2000), or there was a unique peak but this did not correlate with the expected fundamental physiological frequency (for instance, Cardiac: S1_3T_TR_1520, S4_3T_TR_2000; Respiration: S3_3T_TR_1020, S9_3T_TR_700). Filtering the power spectra of 19 runs (23.75%) (for instance, Cardiac: S2_3T_TR_700, S6_3T_TR_1020; Respiration: S2_3T_TR_1520, S7_3T_TR_1020) may have improved the quality of the estimators. Although the main peak was wrongly estimated, filtering around it would have included the expected value in the distribution. Finally, the quality of the power spectrum of 13 runs (16.25%) could likely have been improved because the fundamental physiological frequencies were correctly assessed (for instance, Respiration: S1_3T_TR700, S6_3T_TR1020; Cardiac: S1_3T_TR_2000, S6_3T_TR_1520) and defining a narrower window, by hand, would have reduced the contribution of other nonphysiological contributions to regressors (Respiration: S1_3T_TR_700 and S1_3T_TR_2000).

Standard fMRI studies generally involve the whole brain with conventional protocols as those used in the HCP, e.g., larger in‐plane resolution or no distance factor (see Supplementary Information, Table C‐2). Correlation of PREPAIR‐magnitude and PREPAIR‐phase waveforms with the external signals (Supplementary Information, Table C‐3) are consistent with our strategy to use both phase and magnitude data, that is, PREPAIR can accurately detect physiological fluctuations in the fMRI data in the whole‐brain single subject study. The removal of those fluctuations is also a success as PREPAIR achieves similar proportion of respiratory and cardiac noise removed as RETROICOR‐EXT, and better than PESTICA (Supplementary Information, Table C‐4). PREPAIR also remains the best tool to limit the addition of noise in the whole‐brain fMRI data after regression of the respiratory and cardiac regressors (last row of Supplementary Information, Table C‐4).

Finally, PREPAIR proved to be efficient in improving the variance in regions outside the cerebrum where imaging and the identification of the anatomical structure is challenging. The brainstem, for instance, is involved in disorders affecting autonomic dysfunctions (Brook & Julius, 2000), affective disorders (Paul & Lowry, 2013), migraine (Denuelle & Fabre, 2013), and Parkinson's disease (Braak et al., 2003; Holiga et al., 2015; Tison & Meissner, 2014), but is subject to high levels of physiological noise. The proximity of the brainstem to the fourth ventricle and arteries has made it the focus of effort to reduce the variance of contaminated voxels and improve sensitivity (Beissner et al., 2011; Harvey et al., 2008; Matt et al., 2019) to allow the depiction of quite small nuclei (D'Ardenne et al., 2008; Thompson et al., 2006). This can be achieved without the need for external physiological recordings using an anatomically defined mask of brainstem and ICA (Beissner et al., 2014). Combining this with temporal noise regression would reduce variance further, provided that the physiological noise removal tools did not remove signals of interests or add noise to the data, which would impede the detection of neurally driven responses, as shown by Agrawal et al. (2020) with the method CompCor (Behzadi et al., 2007). Our results have demonstrated that PREPAIR performed similarly to RETROICOR‐EXT and PESTICA, and significantly better than FIX, in the preservation of the integrity of the magnitude signal outside areas affected by physiological noise in the 3 and 7 T studies (see Tables 3 and 4).

**TABLE 3:** Mean and standard deviation of the percentage of physiological noise removed (two first rows) in the physiological bands and power fluctuation (last row) outside these bands, by each method over all subjects and protocols for the 3 T study. PREPAIR was slightly less effective than FIX in decreasing power in the respiratory band but significantly more effective than PESTICA (***p < .001 and similar to RETROICOR‐EXT. Cardiac noise reduction with PREPAIR was significantly less than FIX (*p* < .001). The power in other spectral regions (power fluctuation) with PREPAIR was changed significantly less than the FIX (*p* < .001) and PESTICA (*p* < .01)

3 T	RETROICOR‐EXT	FIX	PESTICA	PREPAIR
% of noise removed: Respiration	32.1 ± 13.7	33.7 ± 16.4	4.8 ± 10.5***	30.0 ± 13.1
% of noise removed: Cardiac	20.7 ± 18.6	33.7 ± 16.7***	6.0 ± 12.3***	20.0 ± 17.8
Power fluctuation (%)	1.4 ± 2.7	28.4 ± 11.8***	3.3 ± 5.7**	1.9 ± 5.0

**TABLE 4:** Mean and standard deviation of the percentage of physiological noise removed (two first rows) in the physiological bands and power fluctuation (last row) outside these bands, by each method over all subjects and protocols for the 7 T study. For respiration, PREPAIR was significantly more effective in decreasing power in the respiration band than FIX and PESTICA (*p* < .001), and similar to RETROICOR‐EXT. Cardiac noise with PREPAIR was comparable to that of RETROICOR‐EXT, and significantly more effective than FIX and PESTICA (*p* < .001). The change in power in other spectral regions (power fluctuation) with PREPAIR was slightly higher to that of RETROICOR‐EXT and PESTICA but significantly more effective than FIX (*p* < .001)

7 T	RETROICOR‐EXT	FIX	PESTICA	PREPAIR
% of noise removed: Respiration	(28.7 ± 17.8)	−17.1 ± 33.6***	−2.0 ± 12.6***	27.3 ± 14.6
% of noise removed: Cardiac	23.6 ± 11.7	−10.1 ± 32.2***	0.0 ± 8.4***	22.3 ± 15.7
Power fluctuation (%)	1.1 ± 3.3	−27.8 ± 23.9***	1.5 ± 5.4	2.5 ± 2.6

## 5 CONCLUSIONS

The proposed physiological noise correction method PREPAIR uses time series phase and magnitude images to identify respiratory and cardiac fluctuations in fMRI, unsupervised and without external recordings. The physiological signals identified by PREPAIR agreed well with those from the respiratory bellows and photoplethysmography and led to a similar quality of correction to a method based on external recordings—RETROICOR‐EXT—and ICA‐based FIX (performed in unsupervised mode) at 3 T, and better than other recording‐free noise correction method PESTICA (performed in unsupervised mode), at both 3 and 7 T.

## CONFLICT OF INTEREST

The authors have no competing interests to declare.

## Supporting information


**APPENDIX S1** Supporting Information

## Data Availability

All source data are publicly available at the Harvard Dataverse (https://dataverse.harvard.edu/dataverse/prepair). The package PREPAIR is available at (https://github.com/daveB1978/PREPAIR).
