# Supplementary material for: Unsupervised physiological noise correction of functional magnetic resonance imaging data using phase and magnitude information (PREPAIR)
Source: Hum Brain Mapp. 2022 Nov 19;44(3):1209–26. doi: 10.1002/hbm.26152 (PMC9875918; doi:10.1002/hbm.26152)
Supplement: Supplementary file 1 — APPENDIX S1 Supporting Information [file HBM-44-1209-s001.pdf]

Supplementary Material

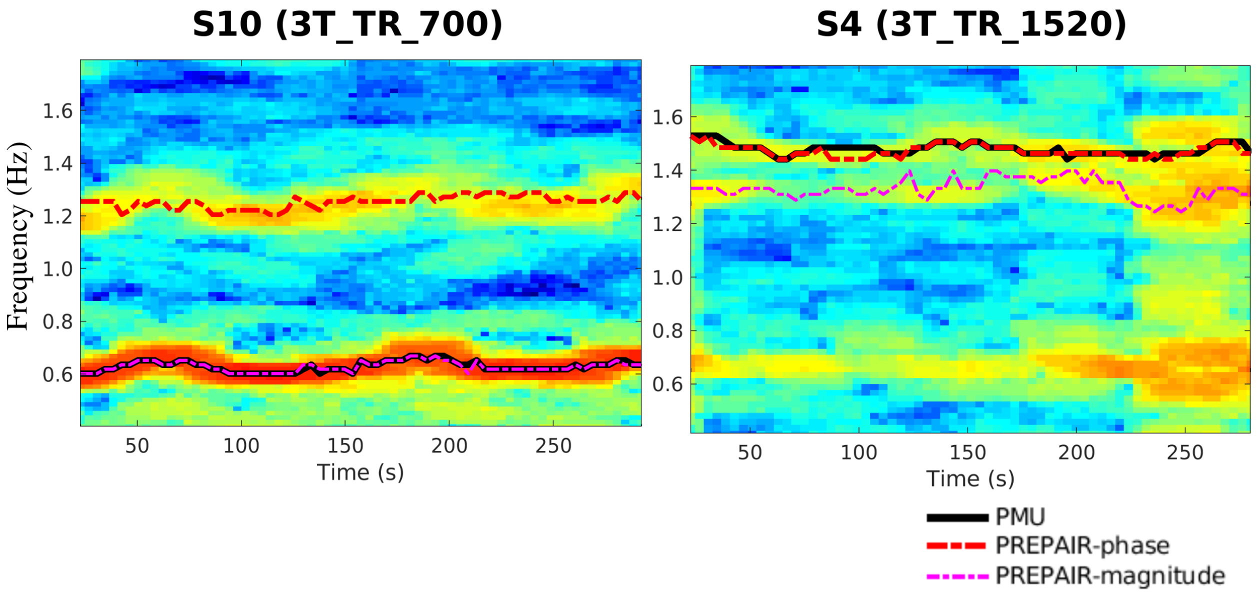

Figure S1: Spectrograms of two subjects with low (left: S10 with a cardiac frequency of ~0.6 Hz) and high (right: S4 with a cardiac frequency of ~1.5 Hz) cardiac dynamics, identified only by PREPAIR-magnitude (magenta) in the first case and only PREPAIR-phase(blue) in the second case. In both cases, the PREPAIR cardiac frequencies estimates overlay well with those identified by the PMU (black).

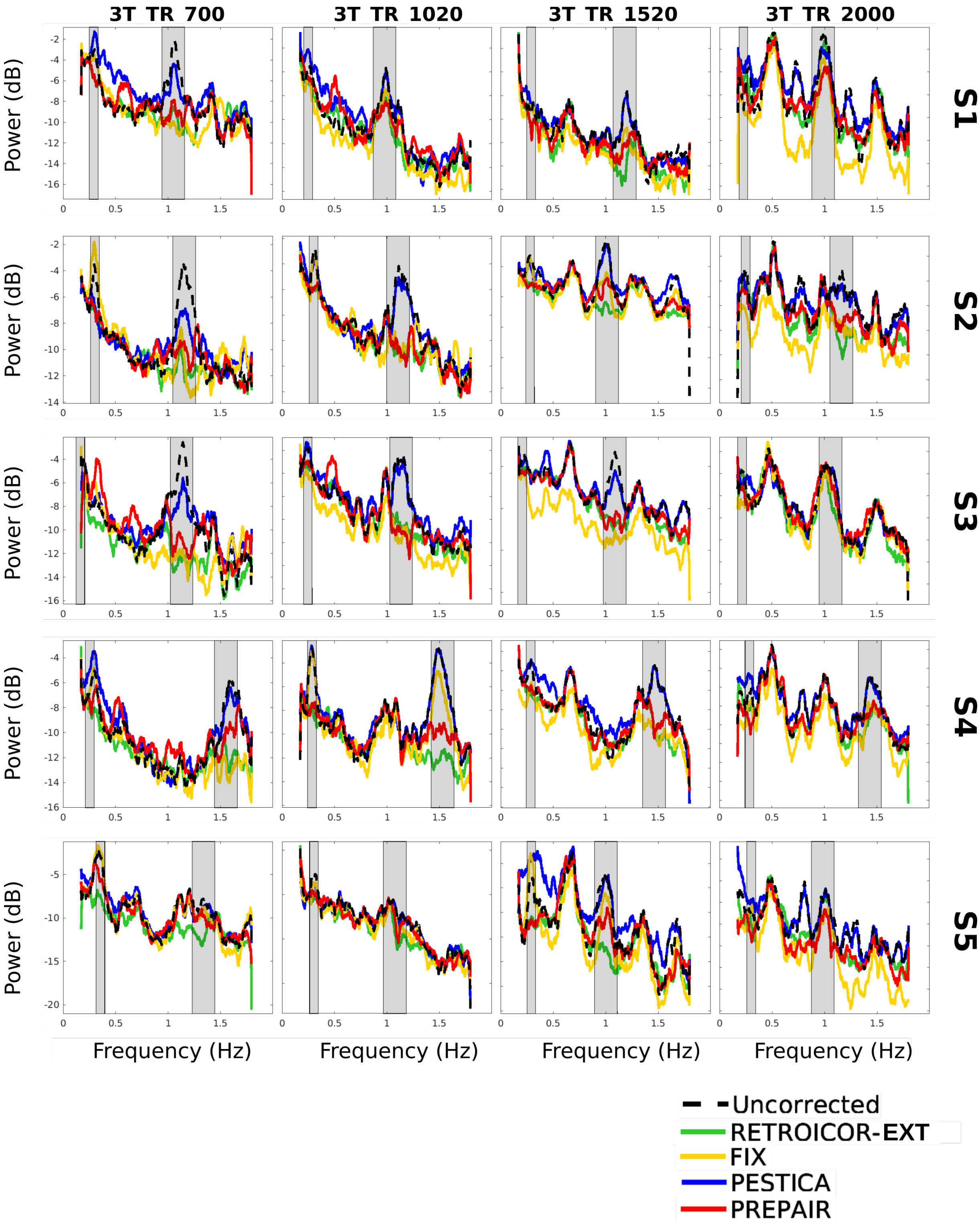

Figure S2: Comparison of the power spectrum of the uncorrected (dashed black line) and corrected (green, yellow, blue, and red lines for RETROICOR, FIX, PESTICA and PREPAIR, respectively) magnitude data for the first five subjects of the 3 T study (rows) for each protocol (columns). Grey boxes are for the location of the 1st harmonic (and 2nd when applicable) of respiratory and cardiac noise. For a better visualization, all frequency distribution were smoothed.

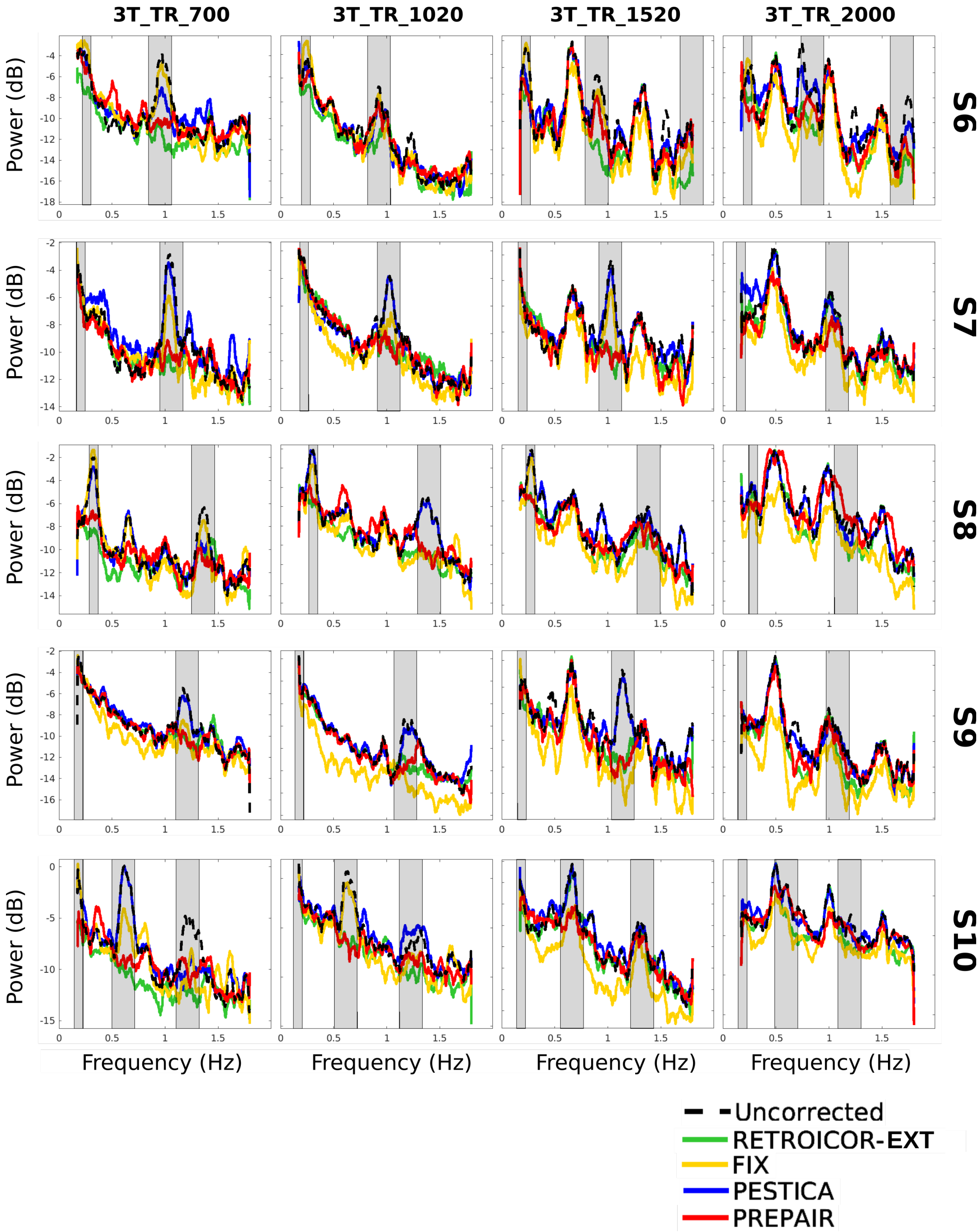

Figure S3: Comparison of the power spectrum of the uncorrected (dashed black line) and corrected (green, yellow, blue, and red lines for RETROICOR, FIX, PESTICA and PREPAIR, respectively) magnitude data for the last five subjects of the 3 T study (rows) for each protocol (columns). Grey boxes are for the location of the 1st harmonic (and 2nd when applicable) of respiratory and cardiac noise. For a better visualization, all frequency distribution were smoothed.

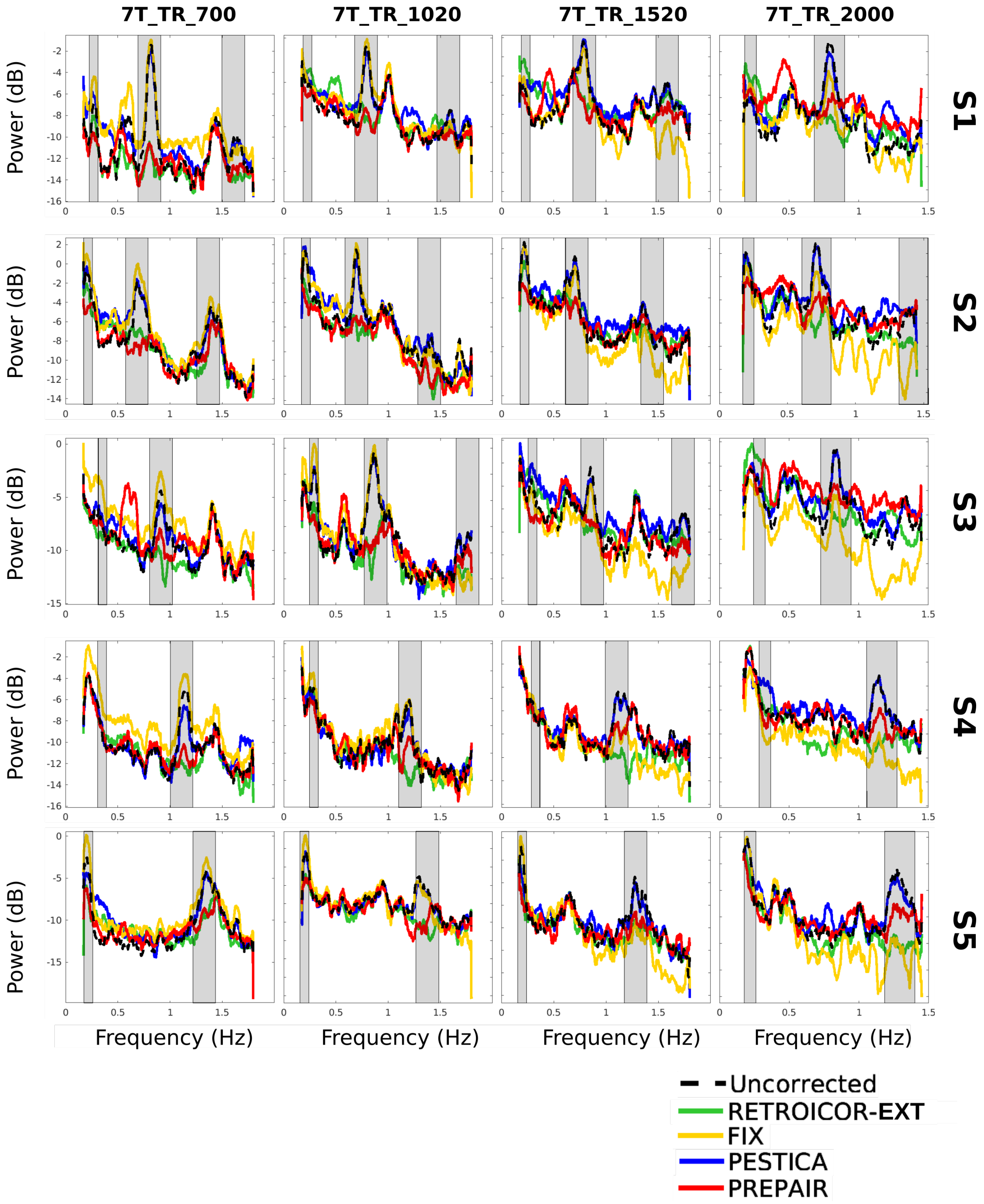

Figure S4: Comparison of the power spectrum of the uncorrected (dashed black line) and corrected (green, yellow, blue, and red lines for RETROICOR, FIX, PESTICA and PREPAIR, respectively) magnitude data for 7 T study (rows) for each protocol (columns). Grey boxes are for the location of the 1st harmonic (and 2nd when applicable) of respiratory and cardiac noise. For a better visualization, all frequency distribution were smoothed.

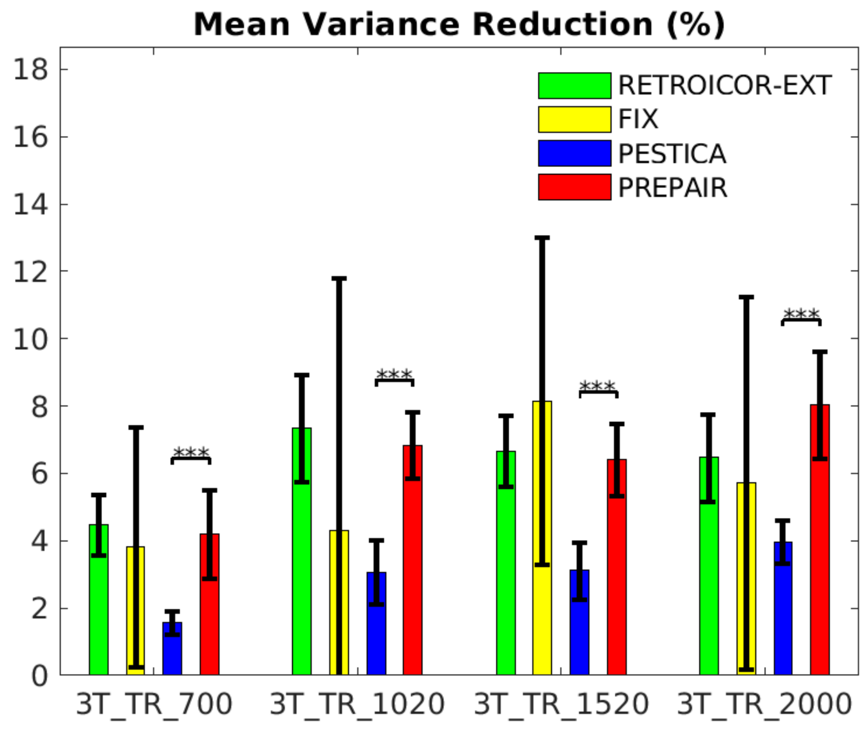

*Figure S5: Variance improvement over all subjects. Because of additional unrelated physiological noise removed in some subjects, FIX would outperform PREPAIR. For all protocols, PREPAIR performed similarly as RETROICOR-EXT and significantly better than PESTICA (***: p<0.001).*

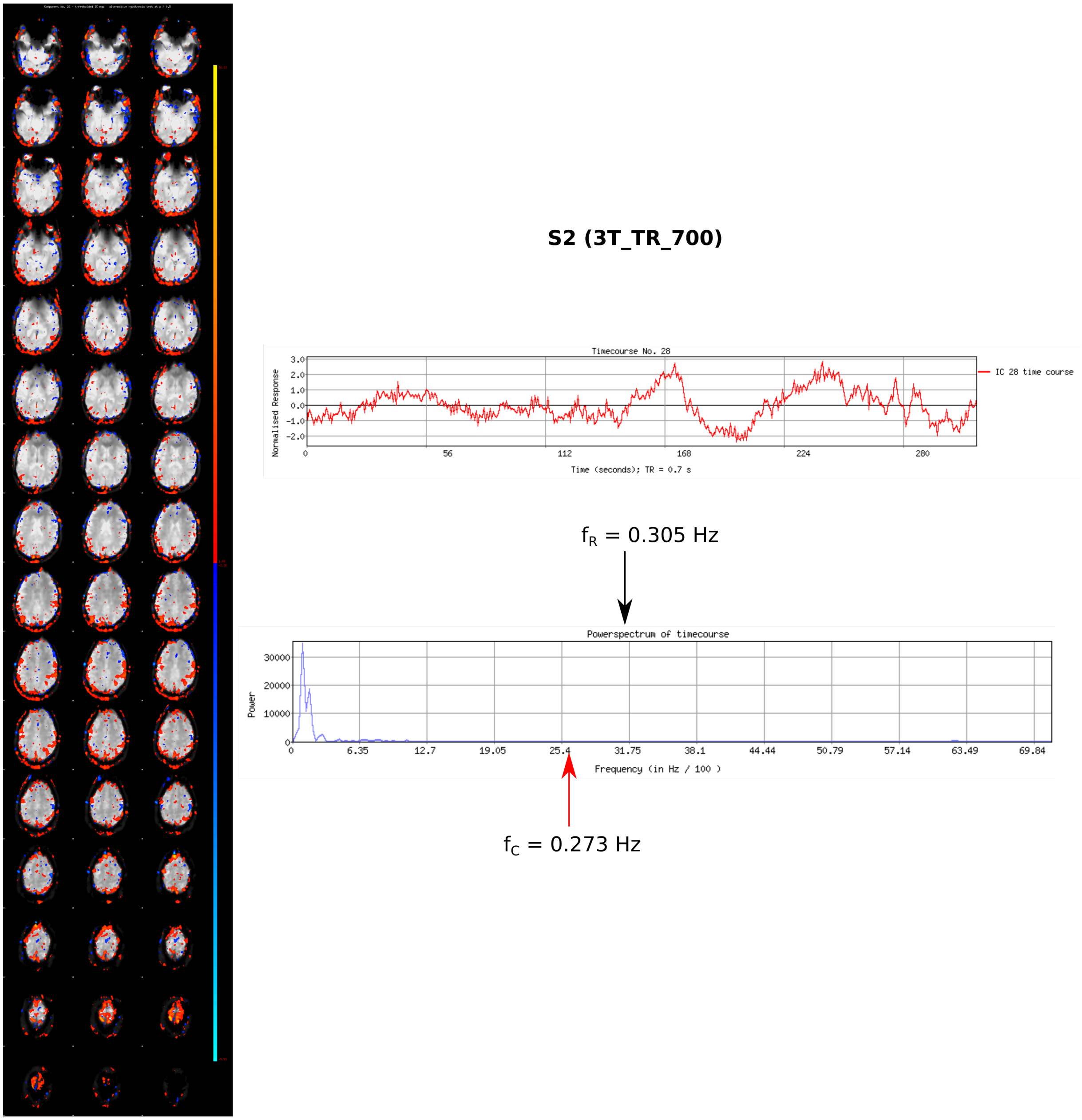

Figure S6: Eye motion-dominated component identified as physiological noise by FIX: an example of those components which contribute to the wide spread of standard deviations in sFigure S5. Fundamental cardiac f_C (aliases) and respiratory f_R derived from the PMU are shown on the power spectra.

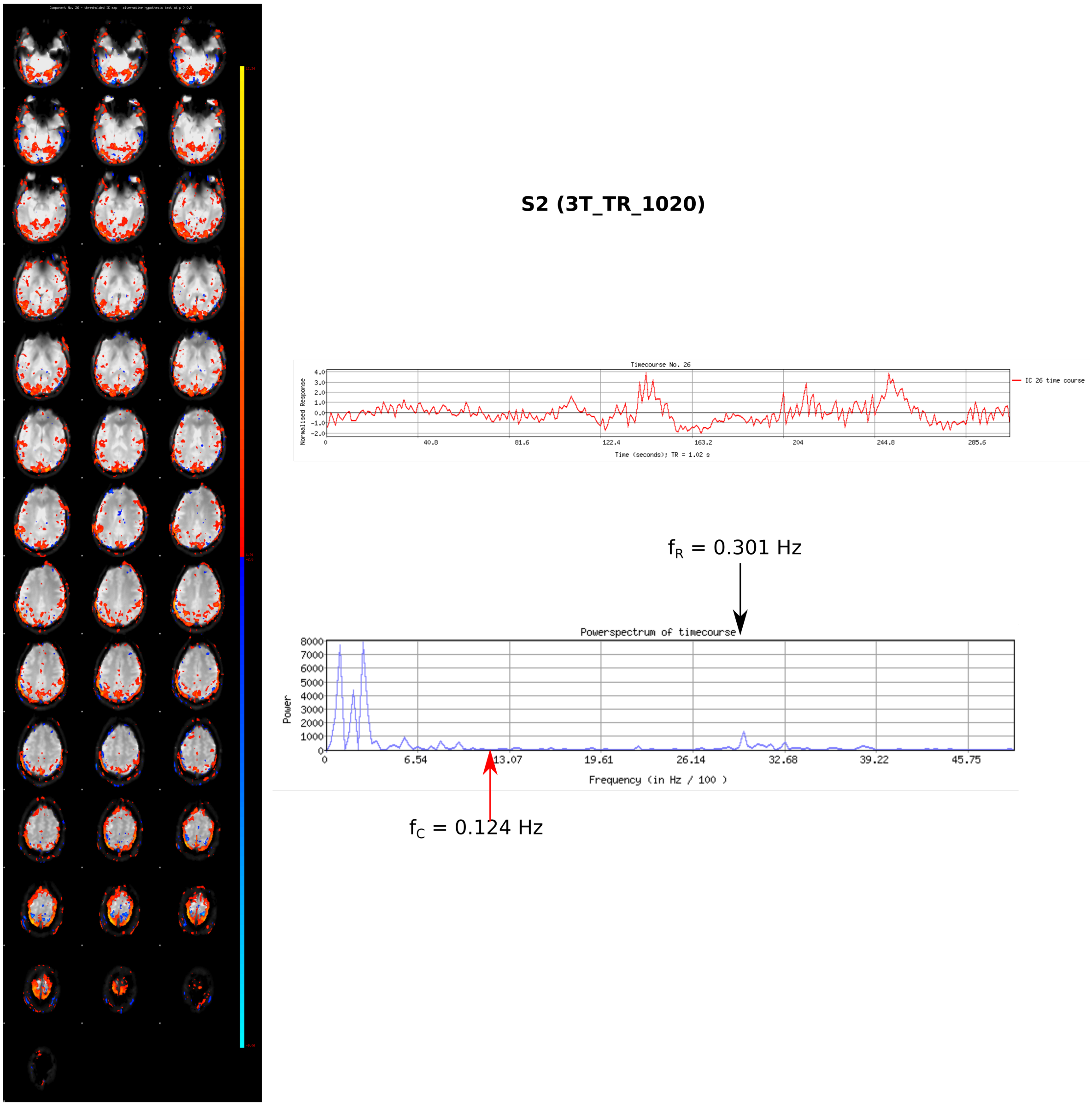

Figure S7: Resting state network-and head motion-related component identified as physiological noise by FIX: an example of a component which contributes to the wide spread of standard deviations in sFigure S5. Fundamental cardiac f_C (aliases) and respiratory f_R derived from the PMU are shown on the power spectra.

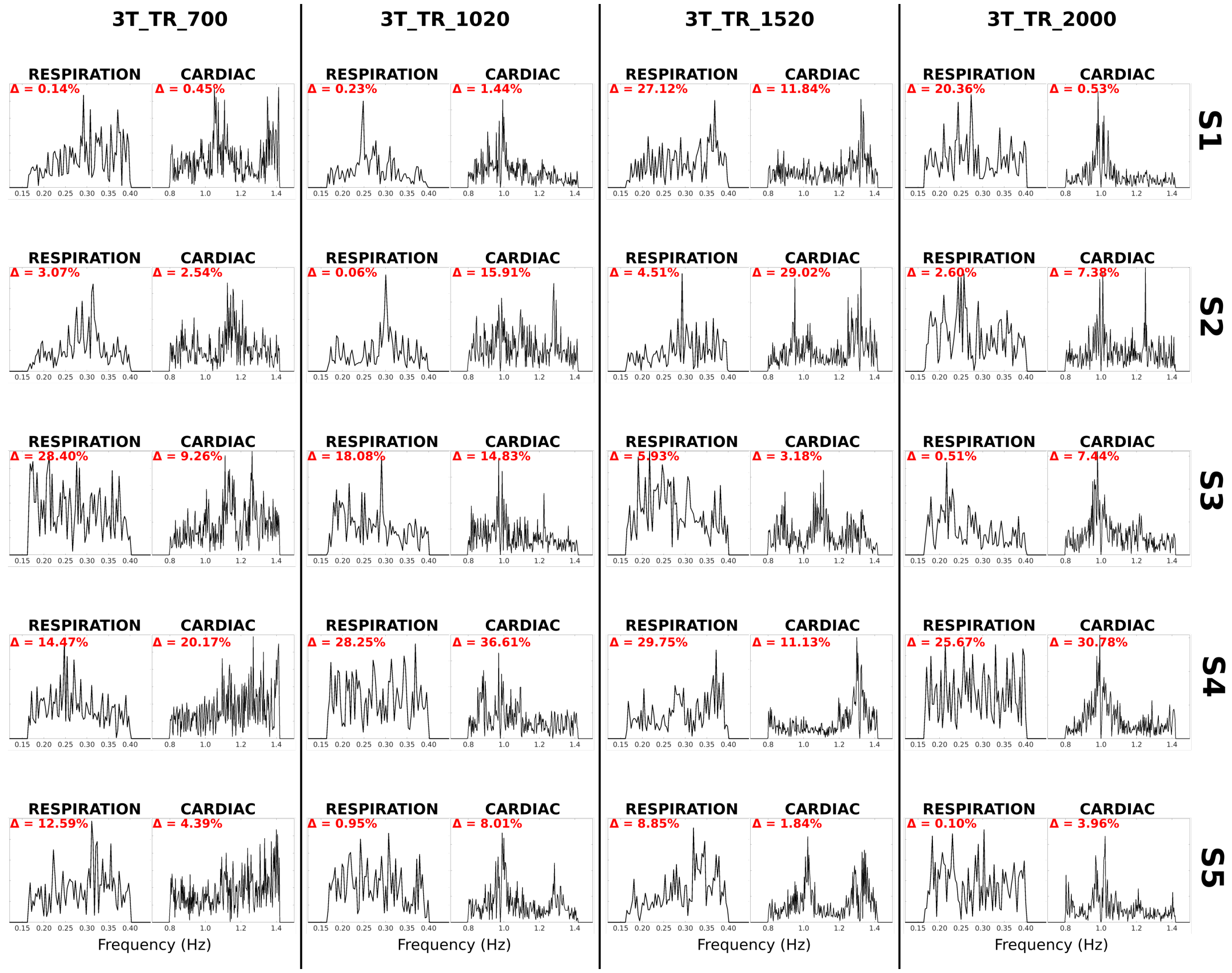

Figure S8: Power spectra of the PESTICA estimators (five first subjects of the 3 T study). Delta values indicate the deviation of the fundamental physiological frequencies from the expected values given by the external recordings.

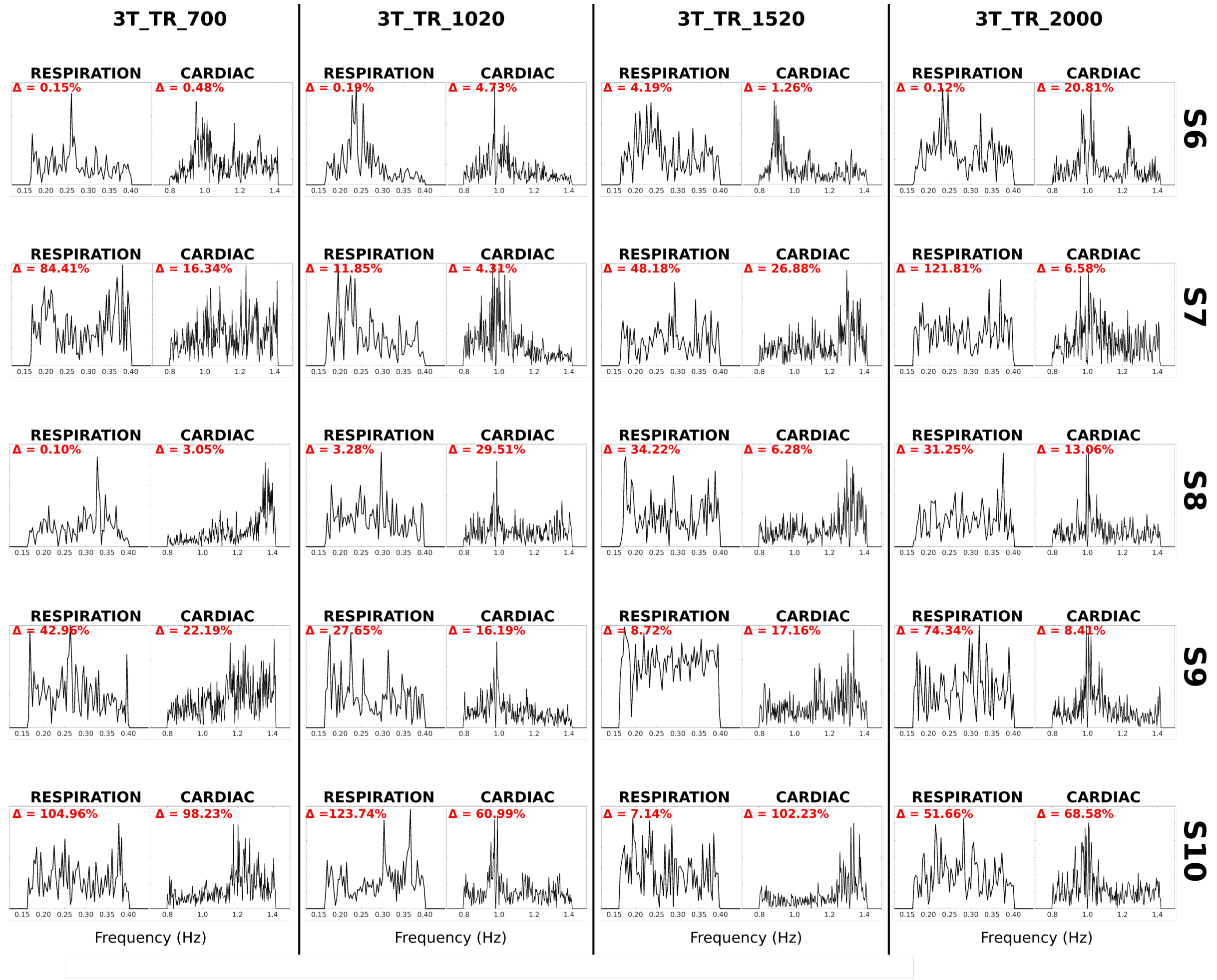

Figure S9: Power spectra of the PESTICA estimators (five last subjects of the 3 T study). Delta values indicate the deviation of the fundamental physiological frequencies from the expected values given by the external recordings.

APPENDIX A. Slice-averaged signals approach versus non slice averaging approach in PREPAIR

In applying PREPAIR to simultaneous multi-slice acquisitions, the signals from slices acquired simultaneously are averaged. The time it takes arterial blood to flow between the slices means that the cardiac signals will not occur simultaneously, however. The highest blood velocities we expect to encounter arise in the Circle of Willis, where they are between 11.7 cm/s (ophthalmic artery) and 58.5 cm/s (middle cerebral artery). This would create a time offset of about 100 – 500 ms for an imaging slab of 12 cm and MB=2. In the following analysis, we assess if there was a significant influence on our method regarding the way regressors were derived i.e. from slice-averaged signals (as in our method see step d in Section 2.3) or without slice averaging. For the approach without

slice averaging, we skipped step b in our pipeline (see Section 2.3) to produce several time series (the number of time series is equal to the value of MB). We then used each time series to derive regressors in the corresponding slice.

We first calculated the correlation of the first component of the cardiac regressors with those obtained with the photoplethysmograph as a function of the time offset between our signal and the one from the external device. We performed that analysis for subject S4 with the protocol 3T_TR_1520; that for which the distance between slices acquired simultaneously was the largest (~ 4.6 cm). With MB = 2 and 34 slices, slices simultaneously acquired are slices (1,18), slices (2,19), etc … and the expected shifts in blood circulation between those slices range between 80 (middle cerebral artery) to 400 ms (ophthalmic artery).

Figure A-10 and Table A-1 show that the time offset between simultaneously-acquired slices S_X and S_Y ranges between 70 and 150 ms, consistent with blood velocities.

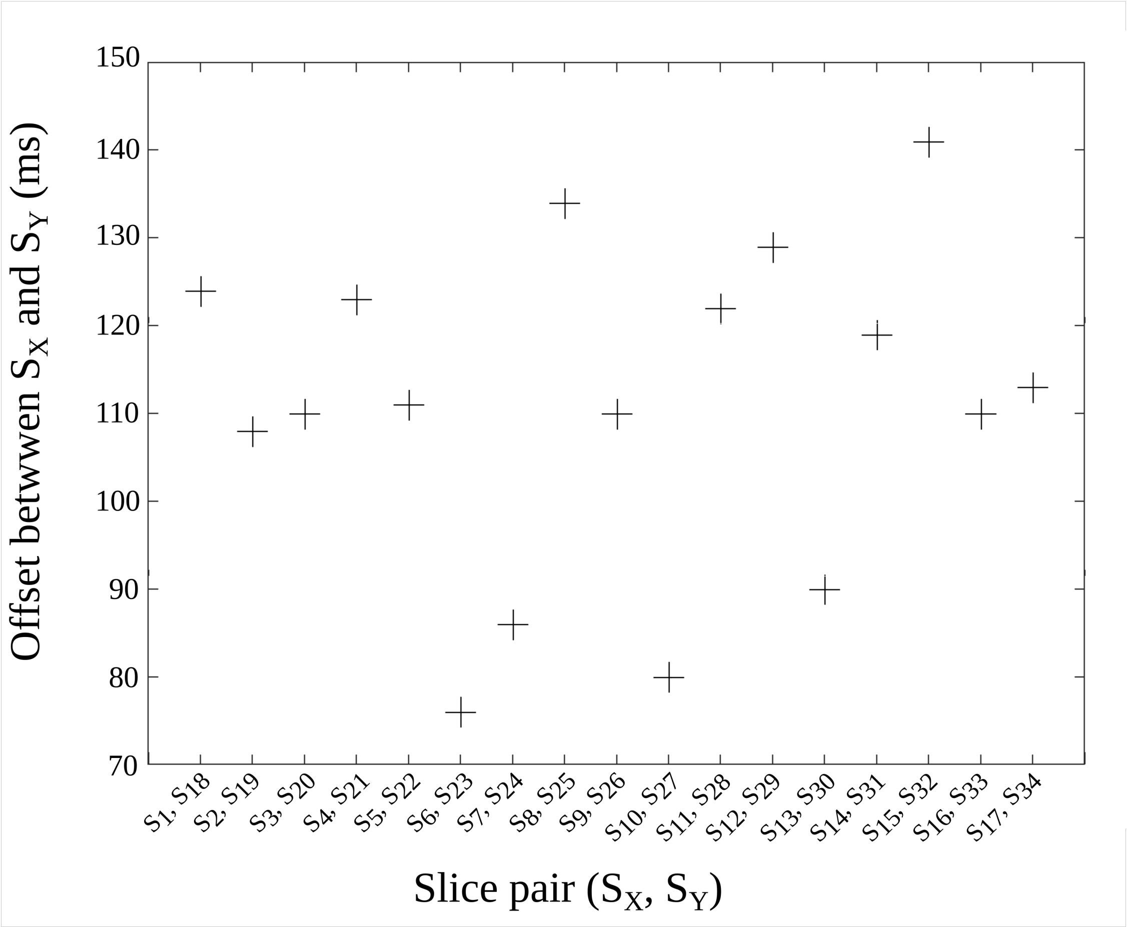

Figure A-10: Time offset (ms) between S_X and S_Y. Values are scattered around a mean offset of 110 ms.

Figure A-10 represents the time lags between regressors of the slices S_X and S_Y simultaneously acquired, ranging from 70 ms to 150 ms (see Table A-1 for exact values) which shows that the cardiac signal in S_X and S_Y do not occur totally simultaneously, although the delay is very low.

S_X - S_Y	S_1 - S_{18}	S_2 - S_{19}	S_3 - S_{20}	S_4 - S_{21}	S_5 - S_{22}	S_6 - S_{23}	S_7 - S_{24}	S_8 - S_{25}	S_9 - S_{26}
Offset(ms)	124	108	110	123	110	76	86	124	110
S_X, S_Y	S_{10} - S_{27}	S_{11} - S_{28}	S_{12} - S_{29}	S_{13} - S_{30}	S_{14} - S_{31}	S_{15} - S_{32}	S_{16} - S_{33}	S_{17} - S_{34}	
Offset(ms)	80	120	129	90	119	141	110	113	

Table A-1: Lag in ms between the pair of simultaneously acquired slices (S_X, S_Y). Minimum lag is 76 ms (S_6, S_{23}) and maximum lag is 141 ms (S_{15}, S_{23}).

As there is a slight delay between signals (and regressors) acquired simultaneously, we calculated – for all subjects and protocols – the proportion of cardiac and respiratory noise removed with regressors obtained without averaging slices and compared that with the proportion of cardiac and respiratory noise removed with regressors obtained without averaging slices. The proportion of

respiratory noise removed was 29.4±13.9% compared to 30.0±13.1% with slice-averaging (see Table C-4) (i.e. statistically essentially the same). Also, the proportion of cardiac noise removed without averaging was 18.5±16.6, and with slice-averaged regressors it was 20.0±17.8 % (see Table C-4), again statistically very similar.

APPENDIX B. Influence of the correlation on image regression

Physiological signals can have different shape, behaviour, and offsets, compared to signal changes we extract from in images. Therefore, they will never perfectly match. When the signals do not match perfectly, less variance related to physiological will be removed compared to signals which match better. This is illustrated Figure B-11 in which we compare cardiac t-maps regressors for two subjects, one with high correlation (top panel: S10_3T_TR700, correlation = 0.96) and the other with much lower correlation (bottom panel: S1_3T_TR1020, correlation 0.59) with the external data. As we can see, although PREPAIR identify similar voxels affected by cardiac fluctuations as RETROICOR, the t values resulting from the regression are much higher when signal better match than when they don’t.

T-maps of the cardiac regressors

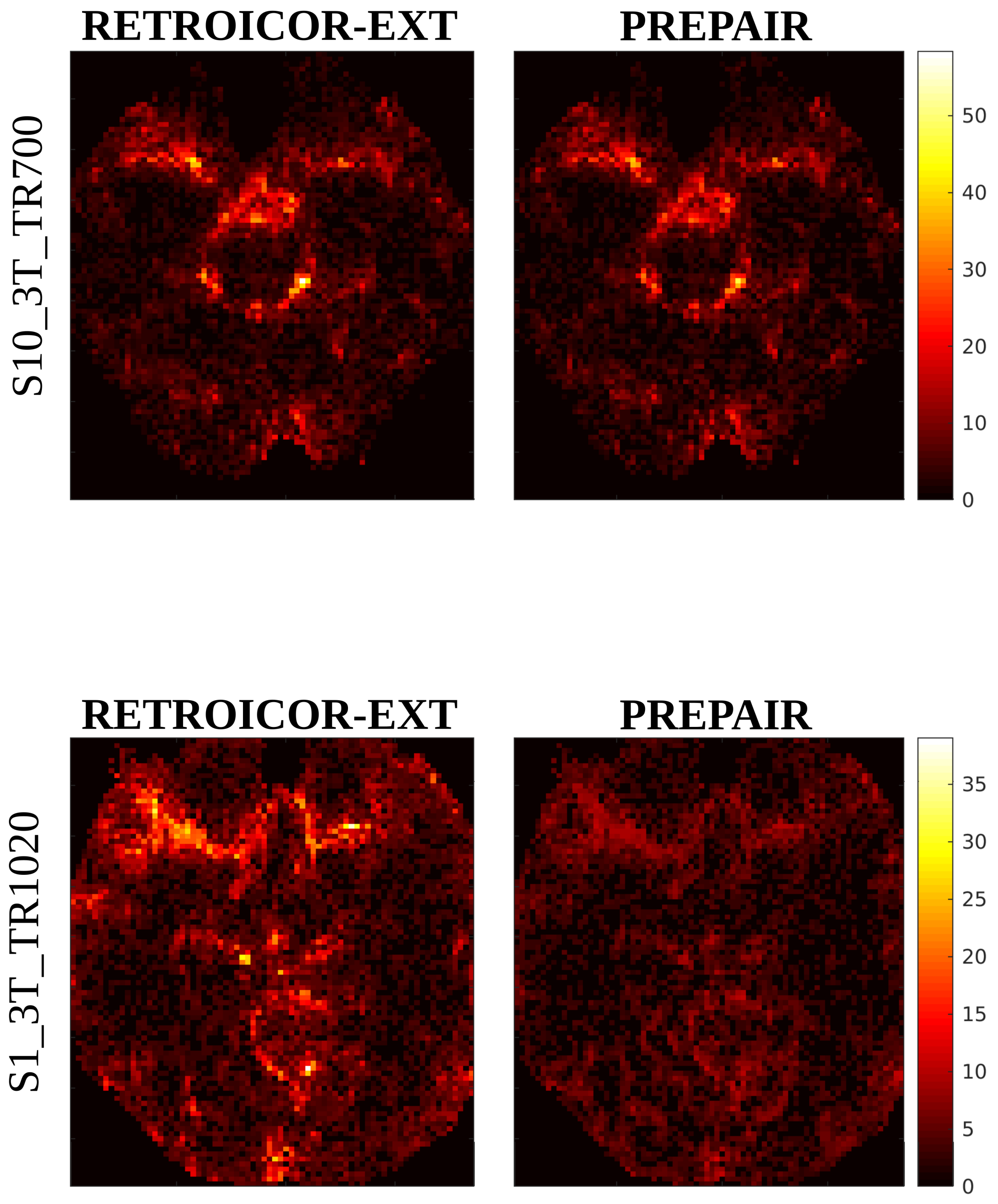

Figure B-11: Comparison of t maps of the cardiac regressors after correction in one slice for subject S10_3T_TR700 (top panel) with a high correlation (0.63) and subject S1_3T_TR1020 (bottom panel) with a lower correlation (0.59). When the correlation is high, t-values of PREPAIR and RETROICOR-EXT are similar (top panel) whereas PREPAIR's t-values are much smaller than that of RETROICOR-EXT when the correlation is low (bottom panel).

APPENDIX C: Application of PREPAIR to isotropic data

In the main manuscript we assess data acquired with quite high spatial resolution and gaps between slices. To test PREPAIR with protocols closer to those of the Human Connectome Project, we scanned one subject on a 3T PRISMA scanner with a 64-channel head coil. Sequential ascending slices were acquired parallel to the AC-PC plane with a matrix size of 112*112, FOV = 220*220 (2.0 mm*2.0 mm in-plane resolution), no distance factor, phase encoding direction: posterior-anterior. Protocols parameters are summarized in Table C-2.

TR [ms]	TE [ms]	FA	MB	N	NR	ST [mm]	GRAPPA
700	28	50	8	56	450	2	2
1020	28	58	4	56	300	2	2
1520	28	67	2	56	200	2	2
2060	28	73	1	39	150	3	2

Table C-2: Acquisition parameters for the whole-brain single subject study. FA = flip angle, MB = multiband acceleration factor, N = number of slices, NR = number of repetitions and ST = slice thickness.

As we can see from Table C-3, the correlation of PREPAIR-phase and PREPAIR-magnitude shows similar trends as in Table 2. With the new protocols, PREPAIR-phase could still accurately detect respiratory fluctuations. For cardiac, PREPAIR-phase correlated much better with the external signals than PREPAIR-magnitude. For TR = 1020 ms, although the correlation is low for PREPAIR-magnitude (0.441), and PREPAIR-phase failed to retrieve cardiac fluctuations, this did not affect the magnitude correction as PREPAIR selected the right time series (PREPAIR-magnitude) for deriving regressors.

		**TR = 700 ms**	**TR = 1020 ms**	**TR = 1520 ms**	**TR = 2060 ms**
**Cardiac**	Correlation: magnitude	0.075	0.441	0.544	0.002
	Correlation: phase	0.744	0.008	0.693	0.722
**Respiration**	Correlation: magnitude	0.917	0.722	0.360	0.051
	Correlation: phase	0.925	0.920	0.888	0.964

Table C-3: PREPAIR-phase and -magnitude correlation with the external signals in the whole-brain single subject study. As in Table 2, PREPAIR-phase modeled respiratory fluctuations better than PREPAIR-magnitude and all PREPAIR-phase signals were used for deriving respiratory regressors. For cardiac, the PREPAIR algorithm selected the signals with highest correlation (PREPAIR-phase for TR = 700, 1520, and 2060 ms; PREPAIR-magnitude for TR = 1020 ms).

Table C-4 shows that PREPAIR is still effective in removing physiological noise. For respiration, PREPAIR removed more noise than RETROICOR-EXT, only for TR = 700 and 1020 ms, and PESTICA, for all TRs. For cardiac, although PREPAIR performed slightly less well that RETROICOR-EXT, it removed much more cardiac noise than PESTICA.

	**TR [ms]**	**RETROICOR-EXT**	**PESTICA**	**PREPAIR**
**Respiratory noise removed**	700	60.4%	42.9%	62.2%
	1020	38.4%	-0.11%	47.8%
	1520	55.9%	31.1%	49.7%
	2060	28.5%	-0.1%	10.1%
**Cardiac noise removed**	700	9.5%	-8.0%	6.2%
	1020	44.9%	21.5%	35.2
	1520	26.1%	19.6%	23.9%
	2060	48.9%	3.0%	32.0%
**Power fluctuation**	700	4.6%	1.1%	1.6%
	1020	3.7%	-12.1%	-1.0%
	1520	3.9%	-10.3%	1.4%
	2060	5.7%	4.3%	3.3%

Table C-4: Percentage of physiological noise removed (two first rows) in the physiological bands (see Section 2.5.2) and power fluctuation (last row) outside these bands by each TR of the whole-brain single subject study. PREPAIR was more effective than PESTICA and similar to RETROICOR-EXT in reducing the variance related to physiological noise. The power in other spectral regions (Power fluctuation) with PREPAIR was changed less than RETROICOR-EXT and PESTICA (except for TR = 700 ms).
